# Emerging natural product development strategies drive the therapeutic potential exploration and clinical translation of hydroxysafflor yellow A

**DOI:** 10.1016/j.jpha.2025.101534

**Published:** 2025-12-23

**Authors:** Haonan Zhu, Xiao Ni, Zhe Yu, Si Liang, Yi Qiu, Jun Huang, Tao Tang, Yang Wang, En Hu, Teng Li

**Affiliations:** a Department of Integrated Chinese Medicine, Xiangya Hospital, Central South University, Changsha, 410008, China; b National Clinical Research Center for Geriatric Disease, Xiangya Hospital, Central South University, Changsha, 410008, China; c NATCM Key Laboratory of TCM Gan, Xiangya Hospital, Central South University, Changsha, 410008, China; d Department of Neurology, Xiangya Jiangxi Hospital, Central South University, Nanchang, 410008, China; e The College of Integrated Traditional Chinese and Western Medicine, Hunan University of Chinese Medicine, Changsha, 410208, China

**Keywords:** Hydroxysafflor yellow A, Natural product medicine, Pharmacological mechanisms, Clinical translation, Strategic innovations

## Abstract

Hydroxysafflor yellow A (HSYA) is a structurally unique quinochalcone di-C-glycoside derived from safflower that exhibits broad-spectrum therapeutic activities, including cardiovascular protective, neuroprotective effects, anti-inflammatory, and antioxidant. As the principal active component in approved clinical injections, HSYA has demonstrated notable efficacy in acute ischemic stroke (IS). Recent breakthroughs in elucidating its biosynthetic pathway have enabled semi-synthetic and *de novo* production, laying the groundwork for industrial-scale application. However, HSYA's clinical translation remains hindered by physicochemical instability, poor oral bioavailability, short half-life, and fragmented mechanistic understanding. Additionally, inconsistent dosing regimens and the lack of in-depth mechanism exploration limit its therapeutic optimization. This review summarizes current advances in HSYA research, including its pharmacology, formulation strategies, and translational barriers. We propose integrative solutions involving nanostructure engineering, systems pharmacology, and artificial intelligence (AI)-assisted target identification to overcome current limitations. Together, these strategies aim to establish HSYA as a generalizable paradigm for advancing natural product-based therapeutics from bench to bedside.

## Highlights

• HSYA exhibits notable cardioprotective, neuroprotective, anti-inflammatory, and antioxidant effects.
• Preparations with HSYA as the main active ingredient have been widely applied in cardiovascular diseases, ischemic stroke, and sepsis.
• Current therapeutic applications and limitations of HSYA are summarized.
• A integrative strategy is proposed to advance the clinical translation of HSYA.

## Graphical abstract

**Figure:**
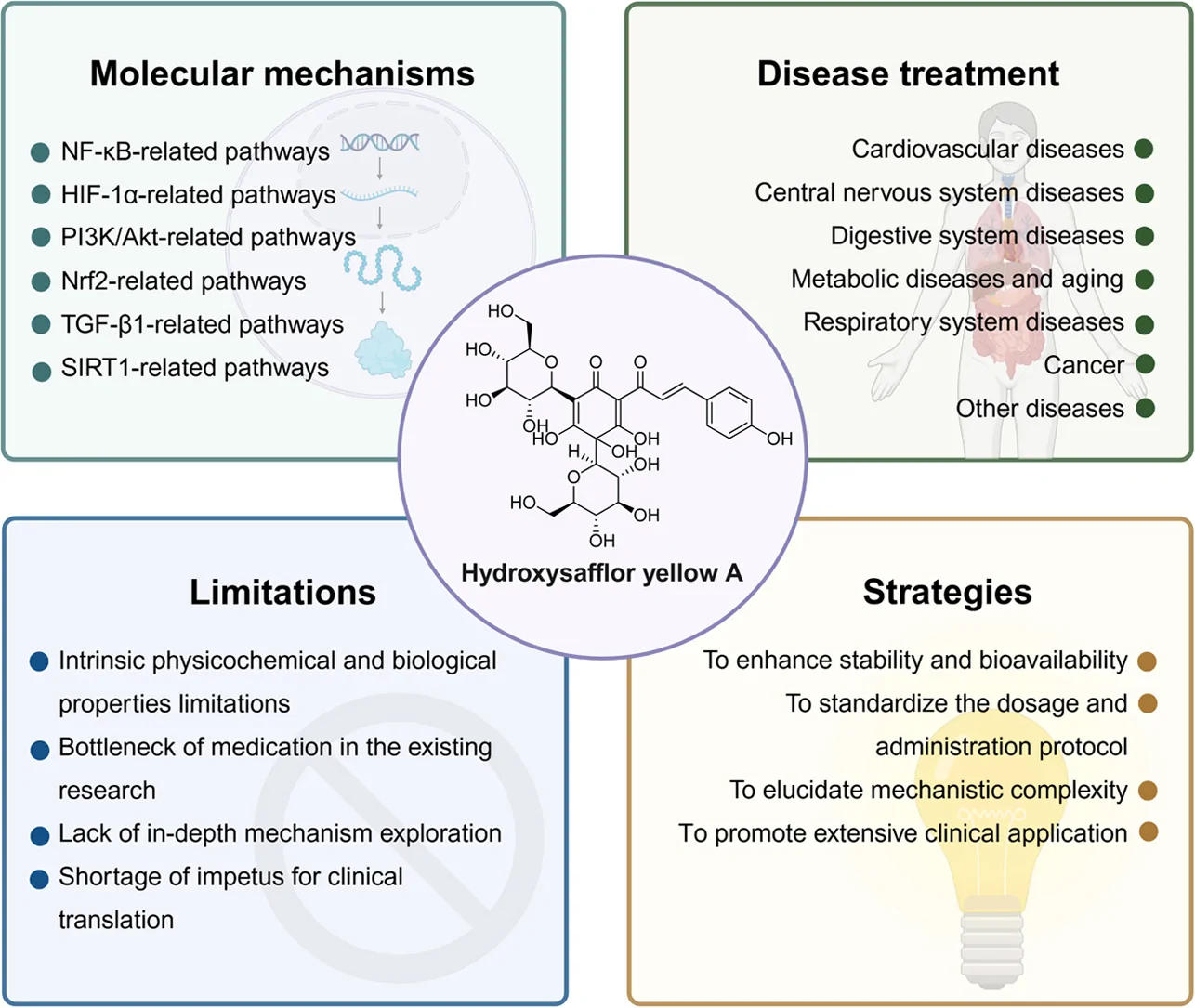

## 1 Introduction

Hydroxysafflor yellow A (HSYA) is a structurally distinctive quinochalcone di-C-glycoside derived exclusively from safflower (*Carthamus tinctorius* L.). Unlike many other flavonoids, HSYA demonstrates excellent aqueous solubility, which facilitates its formulation in injectable preparations and supports clinical use. Over the past decade, HSYA has garnered growing attention for its multi-target pharmacological properties, encompassing cardiovascular protection, neuroprotective, anti-inflammatory, and antioxidant effects. Clinically, HSYA has shown validated efficacy, particularly in the treatment of cardiovascular diseases [1]. It forms the primary bioactive component of widely used Chinese medicinal injections such as Xuebijing and Danhong, which have demonstrated efficacy in sepsis, stroke, and myocardial ischemia [2,3]. Furthermore, phase II and III clinical trials of HSYA injection in acute ischemic stroke (IS) have exhibited significant improvements in neurological outcomes without adverse events reported, indicating a promising therapeutic profile and strong translational potential [4].

The widespread application of drugs requires a stable biosynthetic route and a robust industrial production process. For HSYA, its content in safflower is affected by multiple factors, including geographic origin, flower color, and harvest time [5]. These factors lead to inconsistencies in raw material quality and difficulty in standardization. Although the structure of HSYA was accurately identified over a decade ago [6], its complete biosynthetic pathway remained unclear for a long time. The structurally complex quinochalcone di-C-glycoside nature of HSYA renders total chemical synthesis highly challenging. Elucidating its biosynthetic route is a key question for scalable production. Recent breakthrough work has successfully elucidated the complete biosynthetic pathway of HSYA with four key biosynthetic enzymes [7]. Briefly, flavanone 6-hydroxylase converts naringenin to carthamidin, while chalcone-flavanone isomerase transforms it into carthamidin chalcone. 2-Oxoglutarate-dependent dioxygenase, in coordination with flavonoid di-C-glycosyltransferase, catalyzes the di-C-glycosylation and dearomatization to produce HSYA. Based on these findings, they successfully achieved *de novo* or semi-synthetic production of HSYA in tobacco, yeast, and *in vitro* enzymatic systems. All of these lay a solid foundation for industrial-scale synthesis and significantly accelerate its translational potential.

Nevertheless, the clinical development of HSYA remains hindered by several factors, including fragmented mechanistic insights, inconsistent dosing regimens, and limited clinical studies. Additionally, its unique chemical structure poses challenges related to physicochemical stability, such as photosensitivity and thermal instability. Pharmacokinetically, HSYA exhibits poor oral bioavailability and a short plasma half-life, necessitating frequent administration and complicating long-term patient compliance. In recent years, substantial efforts have been made to overcome these limitations through mechanistic investigations, formulation optimization, expanded clinical evaluation, etc.. However, to date, no comprehensive review has systematically integrated these advances and the remaining translational challenges.

Hence, the present review provides an up-to-date and integrative summary of current HSYA research. We highlight unresolved challenges in its development, and propose an integrative strategy encompassing nanostructure engineering, systemic pharmacology, computer technology guidance, and clinical translation to bridge the gap between preclinical findings and clinical applications. By addressing these challenges, this work aims to position HSYA as a paradigm for the development of natural product-based drugs. Leveraging its multi-pharmacological properties while overcoming inherent limitations through diverse strategies may ultimately accelerate its clinical translation and industrial application.

## 2 Physicochemical and biological properties

### 2.1 Physicochemical properties

**Fig. 1:**
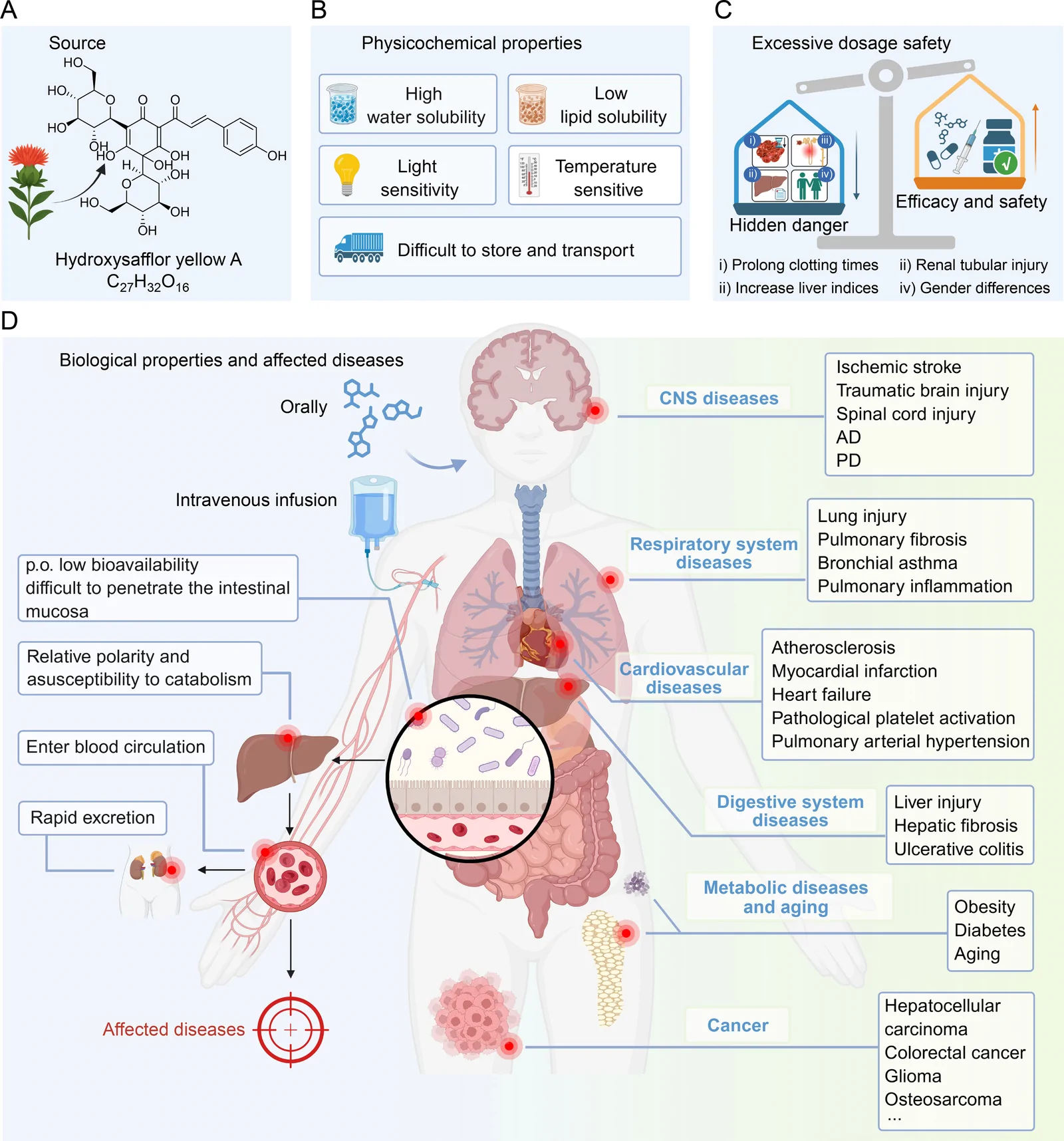
An overview of hydroxysafflor yellow A (HSYA)'s source, properties, safety, pharmacokinetics, and therapeutic uses. (A) The origin of HSYA. (B) Physicochemical characteristics of HSYA. (C) Safety at overdose levels and potential risks associated with HSYA. (D) Administration routes, metabolic pathways, and diseases targeted by HSYA. p.o.: oral administration; CNS: central nervous system. AD: Alzheimer's disease; PD: Parkinson's disease.

HSYA is a bright yellow powder with the chemical formula C_27_H_32_O_16_ and a molecular weight of 612.5 g/mol (Fig. 1A). It exhibits high solubility in water but limited lipid solubility [8]. The phenolic hydroxyl groups render HSYA predominantly protonated in a neutral or alkaline aqueous environment, substantially limiting its ability to cross cell membranes and reducing its bioavailability. Owing to its chalcone-glycoside structure, HSYA is also hygroscopic and photosensitive, necessitating protection from light [5]. HSYA thermally decomposes at temperatures above 60 °C and must therefore be stored under controlled conditions (4 °C or lower). The proposed degradation mechanism involves the colorless glycoside of HSYA forming a conjugated system through bonding with H_2_O [1]. This inherent instability poses considerable challenges for the transportation and long-term storage of HSYA. Moreover, HSYA experiences rapid degradation under highly acidic or alkaline conditions, which undermines its structural stability [9] (Fig. 1B).

### 2.2 Biological properties

Pharmacokinetic research has demonstrated that HSYA exhibits a short half-life and low bioavailability. A clinical trial conducted in healthy Chinese participants who received intravenous administration of HSYA (75 mg/day for 14 days) confirmed rapid systemic absorption, with an elimination half-life ranging from 4.0 to 4.7 h [10]. Another study involving intravenous infusion of HSYA in 36 healthy individuals (18 males and 18 females) corroborated an elimination half-life of approximately 3.912–4.182 h (single-dose from 25 to 75 mg). Continuous administration (50 mg/day for 7 days) slightly prolonged the half-life to 4.414 h compared to 3.912 h after a single 50 mg dose [11]. A clinical finding of a single oral dose (140 mg) in healthy individuals showed rapid absorption with a peak time of 1 h, and a shorter half-life ranging from 2.6 to 3.5 h (mean: 2.9 h) [12]. Despite the limited sample size, these findings suggest that even at a higher oral dose, the half-life remains shorter than that observed with intravenous administration (50 mg). While intravenous administration offers certain pharmacokinetic advantages, the inherent short half-life of HSYA necessitates frequent dosing. It may pose significant challenges for treatment regimens and patient compliance.

Among healthy individuals, HSYA is rapidly absorbed and distributed across multiple organs, including the liver and kidneys. Renal excretion is the primary elimination route [5,13], as confirmed by pharmacokinetic studies in dogs and rats showing rapid urinary clearance of unaltered HSYA [14]. This rapid excretion limits systemic exposure. Therefore, higher doses are typically required to achieve and sustain therapeutic concentrations, partially explaining the dose-dependence of HSYA's therapeutic effect. Although current safety and tolerability analysis suggests that only a few HSYA-related adverse events have been observed, such as prolonged activated partial thromboplastin time [10,11], these events generally resolve spontaneously without intervention. However, the safety implications of higher therapeutic doses remain insufficiently characterized.

Under pathological conditions, such as blood stasis syndrome animal models, the pharmacodynamics of HSYA may differ significantly. Reduced blood flow and impaired circulation prolong HSYA retention in the small intestine and enhance absorption. Compared to the normal rats, these animals exhibit higher uptake and slower elimination of HSYA, indicating altered drug metabolism [15]. Moreover, significant differences were observed in the distribution of metabolites in plasma, bile, urine, and feces following oral administration in rats with blood stasis syndrome, such as an increase in the half-life from 1.13 to 1.35 h. These findings offer valuable insights into the metabolic behavior of HSYA under pathological conditions [16] (Figs. 1C and D).

## 3 Molecular mechanisms of HSYA

**Fig. 2:**
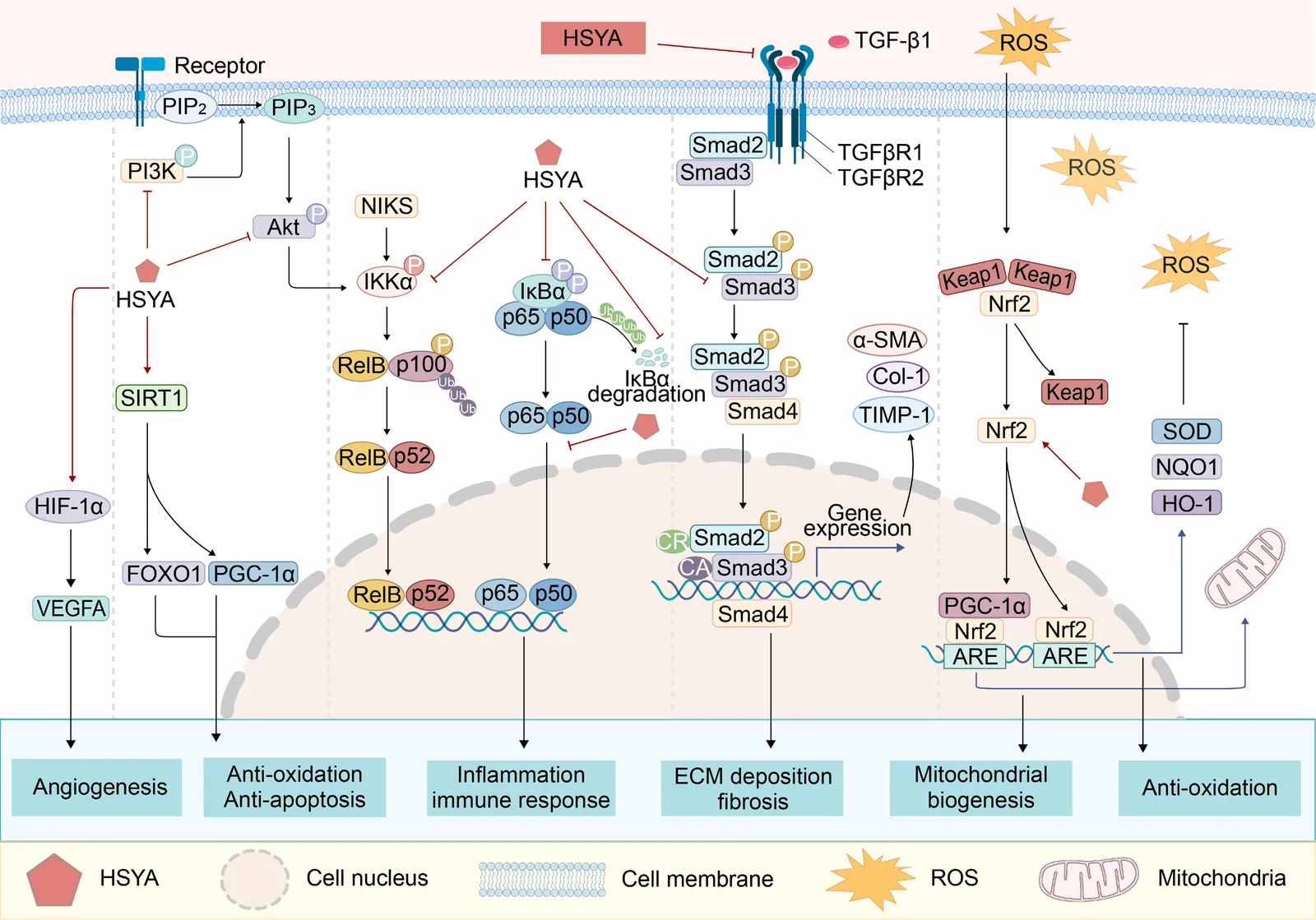
The molecular mechanism of hydroxysafflor yellow A (HSYA) acting on the nuclear factor kappaB (NF-κB)-related pathways, hypoxia-inducible factor-1 alpha (HIF-1α)-related pathways, phosphatidylinositol-3-kinase/protein kinase B (PI3K/Akt)-related pathways, nuclear factor-erythroid 2-related factor 2 (Nrf2)-related pathways, transforming growth factor-beta 1 (TGF-β1)-related pathways, and sirtuin 1 (SIRT1)-related pathways. PIP_2_: phosphatidylinositol 4,5-bisphosphate; VEGFA: vascular endothelial growth factor A; FOXO1: forkhead box O1; PGC-1α: peroxisome proliferator-activated receptor gamma coactivator-1α; NIKS: NF-κB-inducing kinase; IKKα: inhibitor of NF-κB kinase subunit alpha; RelB: v-rel reticuloendotheliosis viral oncogene homolog B; TGFβR1: TGF-β receptor 1; Smad2: SMAD family member 2; CR: co-repressor; CA: co-activator; ECM: extracellular matrix; α-SMA: alpha-smooth muscle actin; Col-1: collagen 1; TIMP-1: tissue inhibitor of metalloprotease-1; Keap1: Kelch-like-epichlorohydrin-associated protein 1; ARE: antioxidant response element; SOD: superoxide dismutase; NQO1: nicotinamide adenine dinucleotide (NAD) phosphate quinone oxidoreductase 1; HO-1: heme oxygenase 1.

HSYA exhibits extensive and multifaceted pharmacological effects. The regulation of many core pathways across various disease models highlights its rich pharmacological effects. Targeting some regulatory mechanisms of these shared pathological processes, such as the nuclear factor kappaB (NF-κB)-related inflammation pathways and the nuclear factor-erythroid 2-related factor 2 (Nrf2)-mediated antioxidant response, as well as specific pathological mechanisms like the anti-fibrotic transforming growth factor-beta1 (TGF-β1)/Smads pathway, HSYA may offer therapeutic benefits in a wide spectrum of complex diseases. Its ability to engage multiple signaling networks provides a mechanistic basis for addressing the multifaceted nature of these pathologies, partially illustrating its potential to co-regulate multiple diseases. Therefore, summarizing its underlying molecular mechanisms is essential for further elucidating its pharmacological effects. Moreover, the selective modulation of certain pathways by HSYA supports its development as either an agonist or an inhibitor, broadening its pharmacological applications (Fig. 2).

The NF-κB pathway encompasses both the canonical and non-canonical pathways [17]. The canonical pathway modulates various expressions of inflammatory factors [18], and the non-canonical pathway regulates immune response and lymphoid organogenesis [19]. HSYA modulates the canonical pathways by reducing the phosphorylation level of IκB-α [20], preventing its cytoplasmic degradation [21], and blocking the nuclear translocation of p65 [22]. In contrast, HSYA regulates the non-canonical pathway by inhibiting IKKα phosphorylation [23], then decreasing inflammatory cytokine production. The hypoxia-inducible factor-1 alpha (HIF-1α) pathway facilitates cellular adaptation to hypoxic environments [24]. By stable binding with HIF-1α, HSYA enhances the transcriptional activity of vascular endothelial growth factor A (VEGFA) and promotes endothelial cell proliferation, migration, and angiogenesis [25]. Through HIF-1α activation, HSYA also increases solute carrier family 7 member 11 (SLC7A11) and glutathione peroxidase 4 (GPX4) to enhance antioxidant capacity [26] and regulate autophagy [27]. The phosphatidylinositol-3-kinase/protein kinase B (PI3K/Akt) signaling pathway regulates cell growth, proliferation, survival, metabolism, and migration [28]. The modulation direction of HSYA appears to be context-dependent. It can suppress the catalytic activity of PI3Kα, block phosphatidylinositol-3,4,5-trisphosphate (PIP_3_) generation [29], and diminish Akt phosphorylation to restrain cell survival and growth [30]. By altering phosphorylation within the PI3K/Akt/mammalian target of rapamycin (mTOR) pathway, HSYA modulates downstream lipid metabolism and inhibits NF-κB activation [23]. However, it may activate this pathway to promote angiogenesis under certain conditions [31]. The Nrf2 pathway serves as a key cellular defense mechanism [32], protecting against oxidative stress via Nrf2 nuclear translocation under reactive oxygen species (ROS) or other stress conditions [33]. HSYA promotes Nrf2 nuclear translocation [34] and upregulates downstream antioxidant targets like nicotinamide adenine dinucleotide (NAD) phosphate quinone oxidoreductase 1 (NQO1) and heme oxygenase 1 (HO-1) [35]. Meanwhile, HSYA enhances peroxisome proliferator-activated receptor gamma coactivator-1α (PGC-1α) activity, with both factors synergistically regulating mitochondrial biogenesis and energy metabolism [36]. The TGF-β1 signaling pathway is the core regulator of the fibrotic process [37], promoting collagen accumulation and extracellular matrix (ECM) deposition while inhibiting ECM degradation [38]. HSYA counters fibrosis by competitively inhibiting TGF-β1/TGF-β receptor 2 (TGFβR2) binding [39], reducing TGF-β/TGFβR signaling pathway activation [40], and suppressing SMAD family member 3 (Smad3) phosphorylation [41]. Sirtuin 1 (SIRT1), a key NAD^+^-dependent deacetylase, critically regulates processes like aging, inflammation, and cell survival [42]. As an activator of SIRT1, HSYA upregulates SIRT1 expression and modulates downstream targets forkhead box O1 (FOXO1) and PGC-1α [43] to alleviate inflammatory and oxidative stress [44]. Additionally, HSYA stabilizes SIRT1 and promotes intracellular retention of high mobility group box 1 (HMGB1), exerting a dual anti-inflammatory effect to inhibit NF-κB activation [45].

## 4 Pharmacological effects and application of HSYA in disease treatment

**Fig. 3:**
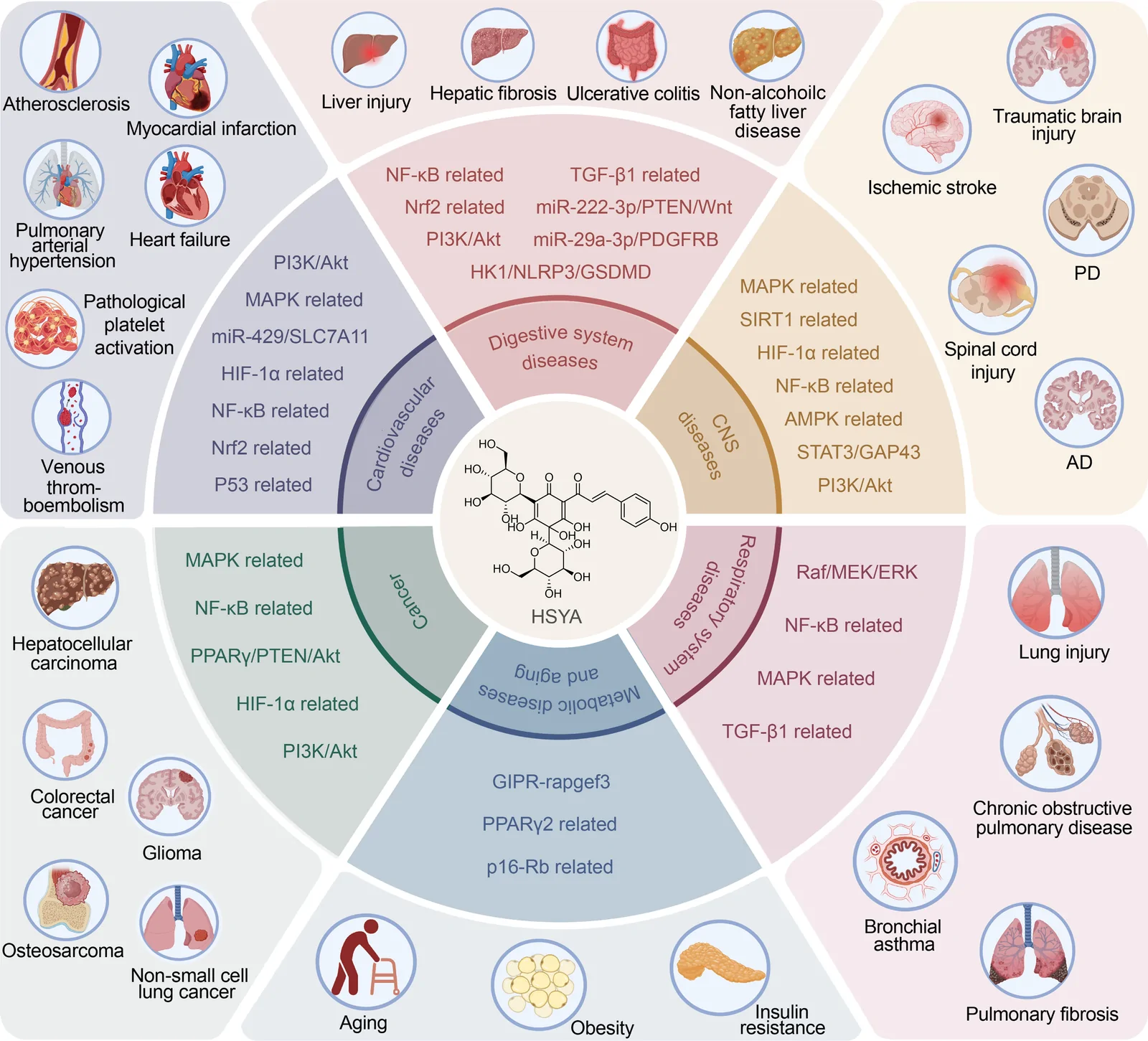
Various diseases are treated with hydroxysafflor yellow A (HSYA). PI3K: phosphatidylinositol-3-kinase; Akt: protein kinase B; MAPK: mitogen-activated protein kinase; miR-429: microRNA-429; SLC7A11: solute carrier family 7 member 11; HIF-1α: hypoxia-inducible factor-1 alpha; NF-κB: nuclear factor kappaB; Nrf2: nuclear factor-erythroid 2-related factor 2; P53: tumor protein 53; TGF-β1: transforming growth factor-beta1; PTEN: phosphatase and tensin homolog; Wnt: wingless/integrated; PDGFRB: platelet-derived growth factor receptor beta; HK1: hexokinase 1; NLRP3: nucleotide-binding oligomerization domain-like receptor family protein 3; GSDMD:gasdermin D; CNS: central nervous system; SIRT1: sirtuin 1; AMPK: 5′-adenosine monophosphate (AMP)-activated protein kinase; STAT3: signal transducer and activator of transcription 3; GAP43: growth-associated protein-43; PPARγ: peroxisome proliferative activated receptor gamma; GIPR: glucose-dependent insulinotropic polypeptide (GIP) receptor; Rb: retinoblastoma; Raf: rapidly accelerated fibrosarcoma; MEK: MAPK kinase; ERK: extracellular regulated protein kinases; PD: Parkinson's disease; AD: Alzheimer's disease.

Due to its different pharmacological properties, HSYA has exhibited significant therapeutic efficacy across a range of diseases. In recent years, research on HSYA has steadily expanded, broadening its applications to include digestive system diseases, metabolic diseases, and aging, among others. These findings show the substantial potential and developmental value of HSYA in therapeutics (Table 1 [20], [21], [22], [23], [25], [26], [29], [30], [31], [35], [39], [40], [41], [43], [45], [46], [47], [48], [49], [50], [51], [52], [53], [54], [55], [56], [57], [58], [59], [60], [61], [62], [63], [64], [65], [66], [67], [68], [69], [70], [71], [72], [73], [74], [75], [76], [77], [78], [79], [80], [81], [82], [83], [84], [85], [86], [87], [88], [89], [90], [91], [92], [93], [94], [95], [96], [97], [98], [99], [100], [101], [102], [103], [104], [105]) and Fig. 3).

**Table 1:** Pharmacological effects and application of hydroxysafflor yellow A (HSYA) in disease treatment.

Disease	Route	Frequency	Dose	Mechanisms	Outcomes	Refs.
Atherosclerosis	i.v.	Four times a week	6.25, 12.5, or 25 mg/kg	Inhibition of PI3K on regulating Akt/mTOR and NF-κB pathway	Inflammation ↓, aortic arches lipid-rich plaque area ↓, the thickness of plaque around the aorta ↓, hepatic steatosis ↓, the number of lymphatic vessels around the plaque of the heart and aortic sinus ↓, leakage of macrophages ↓, and lymphangiogenesis ↓ Body weight ↑ and liver function ↑	[29]
Type 2 diabetes mellitus with atherosclerosis	i.p.	Once daily	2.25 mg/kg	Regulate miR-429/SLC7A11 aixs	Aortic lipid accumulation ↓, atherosclerosis progression ↓, and aortic ferroptosis ↓	[46]
Atherosclerosis	i.g.	Once daily	100 or 200 mg/kg	Inhibit the SphK1/S1P/S1PR3 pathway	Body weight ↓, blood lipids ↓, inflammation ↓, oxidative stress ↓, endothelial damage ↓, aortic plaque area ↓, lipid deposition ↓, aortic lesions ↓, aortic endothelial permeability ↓, and vascular endothelial cells migration ↓ Vascular plaque stability ↑	[47]
Atherosclerosis	–	–	1.2, 12, or 120 μM	Inhibit the TNF-α receptor type 1-mediated classical NF-κB pathway	Adhesion of macrophages to arterial endothelial cells ↓	[48]
MI	–	–	6.25, 12.5, or 25 μM	Regulate the AMPK/NLRP3 pathway	H9c2 cardiomyocyte cytotoxicity ↓, apoptosis ↓, and inflammation ↓ Autophagy ↑	[49]
MI	i.p.	Once daily	2 or 5 mg/kg	Activate Nrf2/NQO1/HO-1 pathway	Cardiac hypertrophy ↓ and oxidative stress ↓	[35]
Acute MI	i.p.	Twice a day	25 mg/kg	–	MI size ↓ and myocardial injury ↓ Cardiac haemodynamics ↑, survival rate ↑, angiogenesis ↑, and HUVECs migration and tube formation ↑	[50]
–	i.v.	Once daily	15, 30, or 60 mg/kg	Upregulate HO-1/VEGFA/SDF-1α signaling cascade	MI Injury ↓, myocardial apoptosis ↓, fibrosis ↓, and left ventricular function impairment ↓ Cardiac function ↑, EPCs mobilization ↑, and myocardial neovascularization ↑	[51]
Myocardial I/R injury	i.v.	Four times in 24 h	20, 40, or 80 mg/kg	Allosteric activator of MDH1	Infarct volume ↓, mitochondrial dysfunction ↓, apoptosis ↓, and ROS production ↓ Cardiac function ↑ and glycolysis and oxidative phosphorylation ↑	[52]
Acute myocardial I/R injury	i.p.	Once daily	5, 10, or 20 mg/kg	Activate the HIF-1α/SLC7A11/GPX4 pathway	Myocardial injury ↓, lipid peroxidation ↓, iron accumulation ↓, ROS levels ↓, and mitochondrial damage ↓	[26]
Myocardial I/R injury	i.v.	Once prior to and once following the operation	4, 8, or 16 mg/kg	Inhibit the activation of NLRP3 inflammasome through regulation of the AMPK/mTOR pathway	Myocardial injury ↓, morphological changes of myocardial tissue ↓, myocardial apoptosis ↓, and inflammatory factors ↓ Autophagy ↑	[53]
Myocardial I/R injury	i.p.	–	5 mg/kg	Inhibit JAK2/STAT1 pathway	Oxidant stress ↓, myocardial infarct size ↓, apoptosis ↓, and inflammation ↓	[54]
Myocardial I/R injury	i.p.	Once daily	7, 14, or 28 mg/kg	Activate HIF-1α-VEGA-Notch1 pathway	MI volume ↓, pathological injury ↓, and ROS level ↓ Blood perfusion volume ↑ and angiogenesis ↑	[25]
Pulmonary arterial hypertension	p.o.	Once daily	220 mg/kg	–	Right ventricular systolic pressure ↓, pulmonary vascular remodeling ↓, collagen deposition ↓, perivascular collagen fibrosis ↓, right ventricular remodeling ↓, and right ventricular fibrosis ↓ Right ventricular function ↑	[55]
Heart failure	–	–	50 μM	Inhibit autophagy	Autophagy ↓, heart failure progression ↓, and ROS level ↓ Cardiomyocytes protection ↑	[56]
Pathological platelet activation	i.p.	Once daily	5 μM	Inhibit the activation of the SRC/PLCγ2/PKC δ/MEK/ERK1/2 axis by upregulating miR-9a-5p	Platelet activation ↓ and platelet aggregation ↓	[57]
Venous thromboembolism	i.p.	–	20 mg/kg	Inhibit the TLR4/NF-κB signaling pathway	NETs-induced endothelial cell ferroptosis ↓, HUVEC apoptotic rate ↓, thrombus length and weight ↓, vein wall thickness ↓, plasma MPO-DNA complexes ↓, plasma cfDNA levels ↓, thrombus formation ↓, neutrophil activation, and oxidative stress ↓	[58]
Thrombosis	–	–	10, 50, or 100 μM	–	Thrombosis formation ↓ Heart RBC levels ↑, blood flow ↑, and blood flow velocity ↑	[59]
Hematopoietic defects	i.p.	–	100 mg/kg	Inhibit the P53 pathway	Cell apoptosis ↓, hematopoietic disorder ↓, oxidative stress ↓, and HSC injury ↓ Neutrophils and macrophages ↑ and leukocytes ↑	[60]
–	–	–	4 or 8 μg/mL	–	Oxidative stress ↓, autophagy ↓ autophagosomes ↓	[61]
Hypertension	–	–	0.1, 1, 10, or 100 μM	–	Relaxing blood vessels ↓	[62]
IS	i.v.	Once daily	5 or 10 mg/kg	MAPK/ERK pathway	Brain impairment ↓, infarct volume ↓, brain edema ↓, and neuropathic damage ↓ NSCs' viability ↑, NSCs to neuronal differentiation ↑, neurological function ↑, blood flow recanalization ↑, neurogenesis ↑, and astrogenesis ↑	[63]
IS	i.v.	30 min before the operation	5 mg/kg	Inhibit the opening of mPTP via the MEK/ERK/CypD pathway	ATP production ↓, mitochondrial damage ↓, ROS levels ↓, infarct volume ↓, neuron damage ↓, mPTP opening ↓, and brain water content ↓	[64]
IS	i.v.	Once daily	1 mL/kg	–	Infarct volumes ↓ Neurological function ↑ and neuronal cells ↑	[65]
IS	i.p.	Once daily	5, 10, or 20 mg/kg	SIRT1-mediated HMGB1/NF-κB pathway	Inflammatory response ↓, neurological scores ↓, brain index ↓, infarct volume ↓, HMGB1 translocation into cytoplasm ↓, and inflammatory ↓ M1 to M2 microglia polarization ↑ and cerebral blood flow ↑	[45]
IS	–	Once following the operation	5 mg/kg	–	Cerebral infarction volume ↓, neurological impairment ↓, hippocampal neuron damage ↓, neuronal ferroptosis ↓, and ferritinophagy ↓	[66]
Stroke	i.p.	Once daily	5 or 20 mg/kg	–	Infarction size ↓ Neurologic behavior ↑, enzymes of phenylalanine metabolism ↑, metabolic increases in phenylalanine ↑, mitochondrial function ↑, and biogenesis ↑	[67]
Cerebral I/R injury	i.v.	Once daily	2, 4, or 8 mg/kg	SIRT1 pathway	Oxidative stress ↓, neuronal apoptosis ↓, neurological deficit ↓, brain infarct volume ↓, and hippocampal neurons structural damage ↓	[43]
–	i.p.	Once daily	50 mg/kg	Inhibit HIF-1α/NOX2 signaling cascades	Brain infarct volume ↓, BBB injury ↓, oxidative stress ↓, microglia infiltration ↓, ROS production ↓, LPS contents in the blood ↓, and lactate production ↓ Brain microvessels integrity ↑	[68]
Stroke	i.v.	–	4, 8, or 16 mg/kg	–	Fear memory ↓ and long term plasticity impairment ↓ Reference memory ↑	[69]
–	–	–	40, 60, or 80 μM	–	ROS level ↓, ferroptosis ↓, parthanatos ↓, and apoptosis ↓	[70]
SCI	i.p.	Once daily	14 mg/kg	Inhibit NF-κB activation	Secondary neuronal death ↓, oxidative stress ↓, inflammatory response ↓, neural apoptosis ↓, tissue edema ↓, BBB permeability ↓, histopathological scores ↓, and apoptosis ↓ Functional recovery ↑	[71]
TBI	i.p.	–	9 mg/kg	Activate AMPK/mTOR pathway	Neuroinflammation ↓ and astrocyte activation ↓ Neuroprotective effects ↑, antiapoptotic effects ↑, and autophagy ↑	[72]
TBI	i.v.	–	10 or 30 mg/kg	–	Antioxidant ↓	[73]
TBI	i.g.	Once daily	13.88 mg/kg	Activate STAT3/GAP43 aixs	Neurological deficits ↓, neuron injury and disarrangement ↓, and inflammatory cell infiltration ↓ Neurogenesis ↑ and axon regeneration ↑	[74]
TBI	i.p.	Once daily	10 or 20 mg/kg	Activate PI3K/Akt/mTOR pathway	Neurological deficits ↓, neuron damage ↓, and neuronal apoptosis ↓ Angiogenesis ↑ and vascular FITC-dextran ↑	[31]
AD	p.o.	Once daily	10 or 30 mg/kg	–	Morphological damage in brain tissues ↓, Aβ aggregates ↓, structural damage to dendritic spines ↓, and glutamate circulation disorder ↓ Synaptic structural plasticity ↑, learning and memory abilities ↑, and short-term memory ↑	[75]
AD	–	–	1, 2.5, 5, 10, or 20 μM	Regulate TREM2/TLR4/NF-κB pathway	Cell viability ↓, morphological changes ↓, and inflammation ↓	[76]
Vascular dementia	i.v.	Once daily	6 mg/kg	–	Neurological function ↑, angiogenesis ↑, synaptic plasticity ↑, and spatial learning and memory ↑	[77]
PD	i.p.	Once daily	2, 4, or 8 mg/kg	Regulate the MAPK inflammatory pathway	Dopaminergic neurodegeneration ↓, dopaminergic neuron apoptosis ↓, and neuroinflammation ↓	[78]
Alcohol-associated liver disease	i.p.	Once daily	2.5 or 7.5 mg/kg	Regulate the PI3K/Akt and STAT3/NF-κB pathways and activate Nrf2/Keap1 pathways	Liver damage ↓, oxidative stress ↓, inflammation ↓, cell toxicity ↓, and hepatocyte apoptosis ↓	[23]
Alcoholic liver disease	Incubate	–	3, 6, or 12 μg/mL	–	Lipid accumulation ↓, oxidative stress ↓, inflammatory response ↓, liver injury ↓, and liver pathological changes ↓ Ethanol catabolism ↑	[79]
Acute liver injury	i.p.	Once daily	10, 20, or 40 mg/kg	–	Wet liver weight ↓, liver index ↓, inflammation ↓, oxidative stress ↓, and liver necrotic area ↓ Liver morphology protection and body mass ↑	[80]
Acute liver injury	i.p.	Once daily	10, 20, or 40 mg/kg	miR-222-3p-PTEN-Wnt axis	Endoplasmic reticulum stress ↓, cell apoptosis ↓, and liver injury ↓	[81]
Non-alcoholic fatty liver disease	i.g.	Once daily	60 or 120 mg/kg	–	Body weight ↓, liver pathology ↓, liver damage ↓, lipid droplets ↓, inflammatory cell infiltration ↓, cell necrosis ↓, fatty liver disease ↓, and *Ruminococcus* ↓ *Turicibacter* levels abundance ↑	[82]
Liver fibrosis	i.g.	Once daily	35, 70, or 140 mg/kg	Inhibit TGF-β1/TGFβR pathway and regulate miR-29a-3p/PDGFRB axis	HSCs proliferation ↓, migration and activation ↓, angiogenesis ↓, liver injury ↓, collagen deposition ↓, and ECM deposition ↓ Anti-fibrotic ↑ and anti-angiogenic ↑	[40]
Hepatic fibrosis	i.p.	Five times a week	5, 10, or 20 mg/kg	–	Liver/body ratio ↓, liver tissue structural damage ↓, collagen fibers deposition ↓, thin fibrous septum ↓, and pseudolobule formation ↓ Lipid metabolism ↑ and protein metabolism ↑	[83]
Hepatic fibrosis	i.p.	Once daily	10 mg/kg	PPARγ/TGF-β1	ROS generation ↓, hepatic fibrogenesis ↓, macro- and microvesicular steatosis ↓, and college synthesis ↓ Liver function ↑, antioxidant enzymes ↑, and liver architecture ↑	[84]
Ulcerative colitis	i.p.	Once daily	30 or 60 mg/kg	Inhibit HK1/NLRP3/GSDMD pathway	Inflammatory responses in macrophages ↓, pyroptosis ↓, glycolysis ↓, weight loss ↓, DAI score ↓, colonic inflammatory infiltration ↓, MPO activity ↓, colonic inflammatory cytokines ↓, and gut microbiota dysbiosis ↓ Colon length ↑ and mucosal integrity ↑	[85]
Ulcerative colitis	i.v.	Once daily	16, 32, or 64 mg/kg	Inhibit TLR4/NF-κB pathway	Colitis symptoms ↓, inflammatory cytokines ↓, inflammatory cells infiltration ↓, and MPO activity ↓ Body weight ↑ and colon length ↑	[86]
Obesity	i.g.	Once daily	200 mg/kg	Modulate the gut microbiota and serum metabolism	Body weight ↓, hepatic fat accumulation ↓, fat excretion ↓, fasting serum glucose ↓, insulin concentrations ↓, insulin resistance ↓, glucose tolerance ↓, inflammation ↓, and serum metabolism aberrations ↓ SCFAs production ↑, intestinal barrier integrity ↑, gut microbial structure ↑, and abundance and function ↑	[87]
Obesity	i.g.	Once daily	250 mg/kg	Inhibit the GIP-GIPR signaling axis	Obesity ↓, abnormal liver function ↓, and mucosal structural changes ↓ Glucose metabolism ↑ and leptin sensitivity ↑	[88]
Obesity	i.g.	Once daily	250 mg/kg	–	Leptin sensitivity ↑	[89]
Obesity	i.p.	Once daily	200 mg/kg	–	Body weight ↓, WAT mass ↓, and WAT percentage ↓ Glucose metabolism ↑ and liver function ↑	[90]
Obesity	–	–	100 mg/L	Activate PPARγ2	Insulin sensitivity ↑	[91]
Diabetic nephropathy	–	–	100 or 200 μM	–	Inflammatory cytokines ↓	[92]
Aging	i.p.	Once daily	25 mg/kg	Suppress the p16-Rb pathway	Oxidative stress ↓ and liver histopathological alterations ↓ Spleen and thymus coefficients ↑ and weight ↑	[93]
Liver aging	i.p.	Once daily	25 mg/kg	–	Liver aging ↓	[94]
Acute lung injury	i.p.	Once following the operation	5 × 10^−5^ mol/kg in 400 μL of saline	Inhibit the Raf/MEK/ERK pathway	Lung histopathologic changes ↓, inflammatory cells infiltration ↓, lung interstitial damage ↓, inflammatory responses ↓, lung wet-to-dry weight ratio ↓, and NET formation ↓ Lung permeability ↑	[95]
Chronic obstructive pulmonary disease	i.p.	The 1st and 14th day	30, 48, or 76.8 mg/kg	Attenuate NF-κB and p38 MAPK signal transduction	Inflammatory cell accumulations in lung BALF ↓, pathological changes ↓, and inflammatory cell infiltration ↓	[96]
Bronchial asthma	–	–	9, 27, or 81 μmol/L	–	Lucifer yellow transmittance ↓, cytosolic calcium level ↓, and inflammatory activation ↓	[20]
Pulmonary fibrosis	i.p.	Once daily	26.7, 40, or 60 mg/kg	–	Morphological changes ↓, lung focal consolidation ↓, lung hydroxyproline content ↓, and inflammatory infiltration ↓ Bleomycin-induced arterial blood gas ↑	[41]
Idiopathic pulmonary fibrosis	–	–	5, 15, or 45 μmol/L	Target TGFβR2	MRC-5 cell proliferation and migration ↓ and ECM deposition ↓	[39]
Pulmonary fibrosis	i.p.	Once daily	35.6, 53.3, or 80 mg/kg	–	Acute lung inflammation ↓, chronic pulmonary fibrosis ↓, left lung indices ↓, inflammatory infiltration ↓, and edema ↓	[97]
HCC	i.p.	Once daily	1.125, 2.25, or 4.5 mg/kg	Block the ERK/MAPK and NF-κB pathway	Tumor weights ↓, cell proliferation ↓, and angiogenesis ↓ spleen/thymus indexes ↑ and apoptosis ↑	[21]
HCC	i.p.	Once daily	1.125 or 2.25 mg/kg	Inhibit p38 MAPK phosphorylation	HepG2 cell viability ↓, proliferation ↓, migration ↓, invasion ↓, and angiogenesis ↓ Apoptosis ↑	[98]
CRC	–	–	25, 50, or 100 μM	Activate PPARγ/PTEN/Akt pathway	HCT116 cell viability ↓, colony formation ↓, proliferation ↓, migration ↓, invasion ↓, and epithelial-mesenchymal transition ↓ Apoptosis ↑	[30]
Glioma	–	–	25, 50, or 100 μM	–	Cell migrate ↓ and invade ↓	[99]
NSCLC	–	–	5, 10, or 20 μM	Inhibit the PI3K/Akt/mTOR and ERK/MAPK pathway	Proliferation ↓, epithelial-mesenchymal transition ↓, migration ↓, invasion ↓, and inflammation ↓ Apoptosis ↑	[100]
Osteosarcoma	–	–	2.5, 5, or 10 μM	Inhibit the HIF-1α/HK2 and SLC7A11 pathway	Cell growth ↓, cell proliferation ↓, Warburg effect ↓, and relative ATP level ↓ Ferroptosis ↑	[101]
Sepsis	i.v.	Once prior to and twice following the operation	60, 120, or 180 mg/kg	–	Peripheral lymphocyte apoptosis ↓ and inflammation ↓ CD4^+^:CD8^+^ T lymphocyte ratio ↑	[102]
Sepsis	i.p.	4 h post-operation	300 mg/kg	Inhibit the JAK2/STAT1 pathway	Multiple organs from CLP-induced damage ↓ Survival rate ↑	[103]
Osteoarthritis	i.p.	Once daily	20 mg/kg	Inhibit NF-κB and MAPK signals	ECM degradation ↓, inflammation ↓, OA development ↓, cartilage surface ↓, cartilage calcification ↓, osteophyte formation ↓, and synovitis ↓	[22]
Endometritis	Injected	–	4 mg/kg	Inhibit TLR2-mediated NF-κB and MAPK pathway	Inflammatory infiltration ↓ and LPO activity ↓ Uterine tissue structure ↑	[104]
Renal I/R injury	i.p.	Once daily	5 mg/kg	Inhibit the CCR4-mediated apoptosis pathway	Apoptosis ↓ and pathological damage ↓ Renal function ↑	[105]

–: no data. ↓: downregulation; ↑: upregulation. i.v.: intravenous; PI3K: phosphoinositide 3-kinase; Akt: protein kinase B; mTOR: mechanistic target of rapamycin; NF-κB: nuclear factor kappaB; i.p.: intraperitoneal injection; miR-429: microRNA-429; SLC7A11: solute carrier family 7 member 11; i.g.: intragastric; SphK1: sphingosine kinase 1; S1P: sphingosine-1-phosphate; S1PR3: S1P-phosphate receptor 3; TNF-α: tumor necrosis factor alpha; MI: myocardial infarction; AMPK: 5′-adenosine monophosphate (AMP)-activated protein kinase; NLRP3: nucleotide-binding oligomerization domain-like receptor family protein 3; Nrf2: nuclear factor erythroid 2-related factor 2; NQO-1: nicotinamide adenine dinucleotide (NAD) phosphate quinone oxidoreductase 1; HO-1: heme oxygenase 1; HUVEC: human umbilical vein endothelial cell; VEGFA: vascular endothelial growth factor A; SDF-1α: stromal cell-derived factor-1 alpha; EPCs: endothelial progenitor cells; I/R: ischemia/reperfusion; MDH1: malate dehydrogenase 1; ROS: reactive oxygen species; HIF-1α: hypoxia-inducible factor 1-alpha; GPX4: glutathione peroxidase 4; JAK2: janus kinase 2; STAT1: signal transducer and activator of transcription 1; p.o.: oral administration; ADP: adenosine diphosphate; SRC: Src proto-oncogene, non-receptor tyrosine kinase; PLCγ2: phospholipase c gamma 2; PKC δ: protein kinase c delta; MEK: mitogen-activated protein kinase (MAPK) kinase; ERK1/2: extracellular signal-regulated kinase 1/2; TLR4: Toll-like receptor 4; NETs: neutrophil extracellular traps; MPO: myeloperoxidase; cfDNA: cell-free DNA; RBCs: red blood cells; P53: tumor protein 53; HSC: hematopoietic stem cell; IS: ischemic stroke; ERK: extracellular regulated protein kinases; NSCs: neural stem cells; mPTP: mitochondrial permeability transition pore; CypD: cyclophilin D; ATP: adenosine triphosphate; SIRT1: sirtuin 1; HMGB1: high mobility group box 1; NOX2: nicotinamide adenine dinucleotide phosphate hydrogen (NADPH) oxidase 2; BBB: blood-brain barrier; LPS: lipopolysaccharide; SCI: spinal cord injury; TBI: traumatic brain injury; GAP43: growth associated protein 43; FITC-dextran: fluorescein isothiocyanate dextran; AD: Alzheimer's disease; Aβ: amyloid beta; TREM2: triggering receptor expressed on myeloid cells 2; PD: Parkinson's disease; Keap1: kelch-like ech-associated protein 1; PTEN: phosphatase and tensin homolog; Wnt: wingless/integrated; TGF-β1: transforming growth factor beta 1; TGFβR: TGF-β receptor; PDGFRB: platelet derived growth factor receptor beta; HSCs: hepatic stellate cells; ECM: extracellular matrix; PPARγ: peroxisome proliferator-activated receptor gamma; HK1: hexokinase 1; GSDMD: gasdermin D; DAI: disease activity index; SCFAs: short-chain fatty acids; GIP: gastric inhibitory polypeptide; GIPR: GIP receptor; WAT: white adipose tissue; Rb: retinoblastoma; Raf: rapidly accelerated fibrosarcoma; BALF: bronchoalveolar lavage fluid; HCC: hepatocellular carcinoma; CRC: colorectal cancer; NSCLC: non-small cell lung cancer; HK2: hexokinase 2; CLP: cecal ligation and puncture; OA: osteoarthritis; LPO: lipid peroxide; CCR4: C−C chemokine receptor type 4.

### 4.1 Cardiovascular diseases

HSYA addresses key pathological processes in cardiovascular diseases, including atherosclerosis, myocardial infarction (MI), myocardial ischemia/reperfusion (I/R) injury, and pulmonary arterial hypertension. By modulating lipid metabolism, oxidative stress, inflammation, and vascular remodeling, HSYA demonstrates therapeutic versatility across diverse preclinical models. Interpreting the cardiac protection mechanism of HSYA and integrating preclinical evidence to delineate its therapeutic landscape is beneficial for proposing clinical optimization strategies.

#### 4.1.1 Atherosclerosis

HSYA exerts multimodal anti-atherosclerotic effects through coordinated modulation of multiple pathways. In apolipoprotein E (*ApoE*)^−/−^ mice, HSYA effectively treated atherosclerosis by inhibiting PI3K and regulating its downstream Akt/mTOR and NF-κB pathways, which in turn reduced inflammation and lymphangiogenesis [29]. This finding confirms that HSYA exerts regulatory effects on multiple downstream pathways of the single target PI3K, playing a synergistic therapeutic role in atherosclerosis. In a mouse model of type 2 diabetes mellitus with atherosclerosis, HSYA ameliorated the pathological progression and lipid peroxidation by modulating the microRNA-429 (miR-429)/SLC7A11 axis within the ferroptosis pathway in endothelial cells [46]. Although the present study employed transcriptome approaches, further investigation is required to elucidate the regulatory mechanism of miR-429 by HSYA. In summary, HSYA not only reduced oxidative stress and inflammation after atherosclerosis but also improved vascular endothelial function by modulating permeability and reducing lipid deposition, which protected against atherosclerosis progression [47]. Similar regulatory effects on inflammation and atherosclerosis were also observed in the tumor necrosis factor alpha (TNF-α)-induced arterial endothelial cell model [48].

#### 4.1.2 MI

HSYA ameliorated the cytotoxicity, apoptosis, and inflammation associated with hypoxia/reoxygenation-induced cardiomyocyte injury by activating the 5′-adenosine monophosphate (AMP)-activated protein kinase (AMPK) and inhibiting the nucleotide-binding oligomerization domain-like receptor family protein 3 (NLRP3) inflammasome activation. Although this mechanism demonstrated the protective effect of HSYA on cardiomyocytes, the exclusive use of *in vitro* models somewhat limited the strength of the evidence [49]. Concurrently, HSYA alleviated myocardial hypertrophy and oxidative stress via activation of the Nrf2/NQO1/HO-1 signaling pathway, showing potential as a treatment for MI [35]. In acute MI models, HSYA upregulated nucleolin expression and promoted endothelial cell migration and angiogenesis, improving cardiac hemodynamics and functional recovery [50]. HSYA promoted endothelial progenitor cell migration to ischemic myocardium, enhancing angiogenesis and myocardial injury resistance post-MI, via the HO-1/VEGFA/stromal cell-derived factor-1 alpha (SDF-1α) pathway. This cascade reaction of cardiac protective signaling has been demonstrated through HO-1 gene knockout experiments and the application of VEGFA and SDF-1α inhibitors, providing relatively robust evidence in support of the hypothesis [51].

#### 4.1.3 Myocardial I/R injury

HSYA provides cardioprotection in myocardial I/R injury through various mechanisms. By binding to cysteine-137 of malate dehydrogenase 1 (MDH1), HSYA ameliorated mitochondrial dysfunction and enhanced glycolysis and oxidative phosphorylation, reducing the area of MI and ultimately preserving cardiac function [52]. It also alleviated myocardial damage and mitigated iron deposition and mitochondrial injury through activating the HIF-1α/SLC7A11/GPX4 pathway [26]. Furthermore, HSYA preserved myocardial morphology and reduced cell death by promoting autophagy via the AMPK/mTOR pathway and decreasing inflammatory factors [53]. In an ischemia-injured myocardium model, HSYA alleviated oxidative stress and inhibited apoptosis, possibly via suppression of the Janus kinase 2/signal transducer and activator of transcription 1 (JAK2/STAT1) signaling pathway [54]. However, this study utilized only a single dose of HSYA (5 mg/kg), and the optimal dosage remains to be determined. In addition, in an acute myocardial I/R model, HSYA further reduced infarction size and promoted angiogenesis by activating the HIF-1α/VEGFA/Notch1 pathway, improving blood flow, and slowing myocardial injury progression [25].

#### 4.1.4 Other cardiovascular diseases

HSYA exerts broad cardiovascular protective effects across multiple pathological contexts. In the pulmonary arterial hypertension model, HSYA significantly attenuated pulmonary vascular and right ventricular remodeling, mitigated fibrosis, and improved right heart function [55]. These findings support the potential application of HSYA in addressing pulmonary arterial hypertension. Regrettably, this study still lacks data regarding the dose-response relationship. In the context of heart failure, HSYA ameliorated autophagy dysregulation by targeting HIF-1α, reducing ROS accumulation, and preserving cardiomyocyte viability under hypoxia, as shown in H9C2 cardiomyocytes and HeLa cells [56]. However, these results have not yet been validated through *in vivo* experimental models. Regarding platelet regulation, HSYA upregulated platelet miR-9a-5p, reducing adrenaline-induced platelet hyperactivation and aggregation. These results suggest its potential in antiplatelet therapy for cardiovascular diseases [57]. HSYA also inhibited the Toll-like receptor 4 (TLR4)/NF-κB pathway, depleting the neutrophil extracellular traps and reducing the length and weight of thrombi. Ultimately, these processes slowed the progression of venous thromboembolism [58]. In zebrafish models, HSYA could also alleviate acute thrombosis induced by phenylhydrazine, improve blood flow velocity, and exhibit a therapeutic effect comparable to that of aspirin at the same dosage [59]. Beyond thrombosis, HSYA protected hematopoietic stem and progenitor cells by inhibiting tumor protein 53 (P53) signaling and alleviating oxidative stress, enhancing their survival and differentiation in zebrafish and mouse models [60]. It highlights the potential of HSYA as a promising therapeutic candidate for hematopoietic disorders. HSYA protected endothelial cells against oxidative stress-induced autophagy and exerted a beneficial vascular protective effect [61]. It could also function as a vascular relaxant by modulating transient receptor potential vanilloid 4 (TRPV4)-dependent Ca^2+^ influx in vascular endothelial cells and promoting protein kinase A (PKA)-dependent endothelial nitric oxide synthase (NOS) phosphorylation, suggesting potential utility in hypertension management [62].

### 4.2 Central nervous system (CNS) diseases

HSYA emerges as a multifaceted neuroprotective agent effective in treating various CNS diseases, including IS, traumatic brain injury (TBI), spinal cord injury (SCI), Alzheimer's disease (AD), and Parkinson's disease (PD). Its therapeutic potential stems from its unique ability to orchestrate cross-talk between oxidative stress, neuroinflammation, and neuronal survival pathways while enhancing synaptic plasticity and blood-brain barrier (BBB) integrity. Unlike its cardiovascular actions, the neuroprotection of HSYA is distinguished by modulating microglial polarization, suppressing ferroptosis, and regulating mitochondrial dynamics. These mechanisms are critical for addressing the complex pathophysiology of neurodegenerative and acute CNS injuries.

#### 4.2.1 Ischemic neurological diseases

HSYA enhanced endogenous neurogenesis in middle cerebral artery occlusion (MCAO) models and alleviated neurological deficits following IS via the mitogen-activated protein kinase/extracellular regulated protein kinases (MAPK/ERK) signaling pathway [63]. HSYA intervention significantly decreased the infarction volume and brain water content following MCAO, providing neuroprotection through the inhibition of mitochondrial permeability transition pore (MPTP) and the MAPK kinase (MEK)/ERK/cyclophilin D (CypD) signaling pathway. As a natural MPTP inhibitor, HSYA shows potential for treating various diseases [64]. HSYA improved neurological function and reduced infarction volume by upregulating epidermal growth factor (EGF) receptor, endothelial NOS, and HIF-1α in MCAO [65]. HSYA also shifted microglia from pro-inflammatory M1 to anti-inflammatory M2 phenotypes post-MCAO/R via the SIRT1/HMGB1/NF-κB pathway, reducing inflammation and promoting neural recovery [45]. HSYA inhibited ferroptosis progression [66], mitigated the elevation in phenylalanine levels induced by the MCAO/R model, and improved mitochondrial function [67]. However, existing studies on ferroptosis and metabolomics have not yet thoroughly explored the underlying mechanisms. HSYA alleviated neuronal apoptosis and neuron damage following the MCAO/R model through the regulation of the SIRT1 pathway [43]. By inhibiting HIF-1α/nicotinamide adenine dinucleotide phosphate hydrogen (NADPH) oxidases (NOX2) cascades, HSYA exhibited a robust protective effect on zonula occludens-1 (ZO-1), offering protection against BBB disruption in photothrombotic stroke models [68]. HSYA enhanced the significant synaptic plasticity and memory function *in vivo* [69] and also protected oxygen glucose deprivation/reperfusion (OGD/R)-damaged cells from ferroptosis and parthanatos *in vitro* [70]. However, further *in vivo* evidence is required to substantiate this effect against parthanatos.

#### 4.2.2 Traumatic neurological diseases

HSYA mitigated secondary neuronal damage in SCI by suppressing NF-κB activation, reducing proinflammatory mediators and oxidative stress markers while preserving blood-spinal cord barrier integrity and enhancing functional recovery [71]. In the TBI model, HSYA alleviated autophagy dysfunction by activating the AMPK/mTOR signaling pathway, while suppressing NLRP3 inflammasome activation. These mechanisms protect neurons from TBI-induced damage and preserve neuronal integrity [72]. Its neuroprotective effect in TBI was further supported by its potent antioxidative capacity [73]. Metabolomics analysis revealed that HSYA modulated arginine biosynthesis and arginine-proline metabolism following TBI. Furthermore, it facilitated neurogenesis and axonal regeneration through activating the STAT3/growth-associated protein-43 (GAP43) axis [74]. Moreover, HSYA also facilitated angiogenesis following TBI via the PI3K/Akt/mTOR signaling pathway. This study employed a rigorous randomization method and incorporated rescue experiments in conjunction with PI3K inhibitors, which enhanced the validity and reliability of the experimental findings [31].

#### 4.2.3 Neurodegenerative diseases

HSYA ameliorated glutamate metabolic disorder, reduced amyloid beta (Aβ) protein accumulation, and enhanced synaptic plasticity in the Aβ_1−42_-induced AD model. These effects contribute to the effective protection of neurons and the long-term restoration of memory function, demonstrating promising therapeutic potential [75]. HSYA alleviated chronic neuroinflammation in BV-2 cells induced by Aβ_1−42_ through modulating the triggering receptor expressed on myeloid cells 2 (TREM2)/TLR4/NF-κB pathway. It requires further validation in animal models to confirm their applicability and reliability *in vivo* [76]. HSYA exhibits neuroprotective effects in animal models of vascular dementia by enhancing synaptic plasticity and ameliorating spatial learning and memory functions [77]. Through mitigating apoptosis and neuroinflammation, HSYA attenuated dopaminergic neurodegeneration and protected against neuronal damage via regulating the MAPK inflammatory signaling pathway in 6-hydroxydopamine-induced PD mouse model [78].

### 4.3 Digestive system diseases

In recent years, numerous studies have highlighted the notable therapeutic potential of HSYA in treating disorders of the digestive system, particularly liver and intestinal diseases. This efficacy is primarily attributed to its ability to simultaneously modulate key pathological processes. Findings from various experimental models suggest that HSYA exerts its effects through multiple molecular pathways, contributing to the restoration of tissue homeostasis and barrier integrity. This section summarizes the mechanisms and molecular targets underlying the protective effects of HSYA on the liver and intestines, which facilitates a more comprehensive understanding of its influence on digestive system disorders.

#### 4.3.1 Liver-related diseases

HSYA effectively treated alcohol-associated liver disease by targeting inflammation, oxidative stress, and apoptosis. It achieved these effects by inhibiting PI3K/Akt and STAT3/NF-κB pathways to slow liver damage and activating the Nrf2/Kelch-like-epichlorohydrin-associated protein 1 (Keap1) pathway to enhance antioxidant defense [23]. Its benefits extend to non-mammalian models. As shown in ethanol-exposed zebrafish, HSYA alleviated oxidative stress, reduced lipid accumulation, and suppressed inflammatory responses at the messenger RNA (mRNA) level. Key targets of HSYA intervention have been identified and validated, including sterol regulatory element binding transcription factor 1 (SREBF1), fatty acid synthase (FASN), and acetyl-CoA carboxylase alpha (ACACA) [79]. In 50% carbon tetrachloride (CCl_4_)-induced acute liver injury, HSYA ameliorated histopathological alterations and restored hepatic function through coordinated anti-inflammatory and antioxidant pathways at the protein level, including TNF-α, interleukin-1 beta (IL-1β), and superoxide dismutase (SOD). Transcriptome sequencing further elucidated additional potential genes, like thymidine phosphorylase (*Tymp*) and fatty acid binding protein 7 (*Fabp7*), might mediate its hepatoprotective effects [80]. Meanwhile, HSYA might protect against acute liver injury by reducing endoplasmic reticulum stress and regulating the miR-222-3p/phosphatase and tensin homolog (PTEN)/wingless/integrated (Wnt) pathway, offering a therapeutic effect similar to silibinin [81]. Furthermore, by leveraging its antioxidant and anti-inflammatory properties at both the protein and mRNA level, HSYA might slow the pathological progression of non-alcoholic fatty liver disease by modulating gut microbiota and amino acid metabolism [82].

Hepatic fibrosis is key to liver injury progression, and HSYA is also seen as a natural compound with potent anti-fibrotic properties. Through the regulation of the miR-29a-3p/platelet-derived growth factor receptor beta (PDGFRB) axis, HSYA ameliorated liver fibrosis and pathological angiogenesis, decreased ECM deposition, and reduced the abnormal proliferation of endothelial cells [40]. Following CCl_4_-induced liver injury, it alleviated liver function impairment and reduced lipid peroxidation, protecting hepatocytes and attenuating fibrosis [83]. The anti-fibrotic effect of HSYA was further linked to the regulation of the peroxisome proliferative activated receptor gamma (PPARγ)/TGF-β1 pathway, inhibition of ROS production, and reduction in collagen deposition [84].

#### 4.3.2 Intestinal-related diseases

HSYA protects the intestinal tract by restoring intestinal barrier function, modulating inflammatory responses, and regulating the intestinal microbiota. Given that microbiota regulation is frequently associated with the management of other concurrent conditions, this part will not be discussed in detail. In ulcerative colitis, HSYA blocked NLRP3/gasdermin D (GSDMD)-mediated pyroptosis by downregulating hexokinase 1, preserving intestinal barrier integrity and partially rebalancing dysbiotic gut microbiota [85]. Concurrently, HSYA inhibited the TLR4/NF-κB pathway to attenuate colonic inflammatory infiltration and tissue damage [86].

### 4.4 Metabolic diseases and aging

HSYA serves as a multifunctional regulatory agent in metabolic processes and age-related pathological conditions. HSYA demonstrates promising therapeutic potential in combating obesity through multi-target modulation of metabolic dysregulation and adipose tissue homeostasis. Emerging evidence highlights its capacity to suppress fat accumulation, enhance insulin sensitivity, and restore intestinal barrier integrity, positioning HSYA as a novel candidate for the intervention of metabolic diseases. In addition to the aforementioned neurodegenerative diseases, HSYA has demonstrated therapeutic potential in other age-related conditions.

#### 4.4.1 Metabolic diseases

Oral administration of HSYA significantly decreased body weight in high-fat diet (HFD) mice by decreasing hepatic lipid accumulation and increasing fecal fat excretion. This intervention also alleviated inflammation and insulin resistance while preserving intestinal barrier integrity via upregulating the tight junction protein ZO-1. This regulatory effect was associated with HSYA-induced modulation of the gut microbiota [87]. HSYA further combated obesity by modulating the obesity-promoting factor glucose-dependent insulinotropic polypeptide (GIP) via a dual mechanism. In detail, it reduced serum GIP levels by inhibiting its production and secretion in the intestine and suppressed GIPR-rap guanine nucleotide exchange factor 3 (*Rapgef3*) signal transduction in both the hypothalamus and adipose tissue. These two mechanisms synergistically attenuated hyperleptinemia and conferred hepatoprotective benefits. Furthermore, oral administration of HSYA provided superior therapeutic efficacy against the pathological changes compared to intraperitoneal injection [88]. This may be attributed to the modulation of the gut microbial structure by oral administration of HSYA. Another study indicated that improvement in leptin sensitivity precedes body weight reduction in HFD models, suggesting its primary role in resetting metabolic homeostasis rather than secondary weight loss effects [89]. Additionally, HSYA alleviates obesity-associated oxidative stress by upregulating antioxidant enzymes, protecting hepatocytes from oxidative damage [90]. In 3T3-L1 adipocytes, HSYA might enhance the expression of PPARγ2, improving insulin sensitivity and adipocyte function and further contributing to its anti-obesity efficacy [91].

Beyond obesity, HSYA has shown therapeutic effects on other metabolic disorders, including diabetes. A comprehensive review has summarized its intervention mechanisms in diabetes [13]. HSYA also demonstrated considerable promising potential in managing diabetic complications such as diabetic nephropathy [92].

#### 4.4.2 Aging

HSYA ameliorated D-galactose-induced liver aging in mice by mitigating pathological alterations and oxidative stress, potentially by inhibiting the p16-retinoblastoma (Rb) signaling pathway [93]. Integrative analyses combining network pharmacology and transcriptomics further suggested that HSYA may exert anti-aging effects through interactions with heat shock protein 90 alpha family class A 1 (HSP90AA1), ATPase sarcoplasmic/endoplasmic reticulum Ca^2+^ transporting 1 (ATP2A1), and NOS1. However, the pathological features of liver aging have not been fully shown, and its therapeutic efficacy requires further investigation [94].

### 4.5 Respiratory system diseases

HSYA also demonstrates significant therapeutic potential in the treatment of respiratory diseases. In pulmonary inflammation, HSYA mitigated lipopolysaccharide (LPS)-induced acute lung injury by suppressing the rapidly accelerated fibrosarcoma (Raf)/MEK/ERK pathway and modulating neutrophil extracellular trap formation, effectively reducing alveolar edema and inflammatory cell recruitment [95]. In the chronic obstructive pulmonary disease model, HSYA could effectively alleviate pulmonary inflammation and mitigate lung tissue damage. This effect may be attributed to the inhibition of NF-κB and p38MAPK signal pathways [96]. In human small airway epithelial cells, HSYA attenuated platelet activating factor (PAF)-induced inflammatory activation by inhibiting NF-κB and MAPK signaling pathways, suggesting its potential utility in bronchial asthma management [20].

HSYA treatment attenuated bleomycin-induced pulmonary fibrosis by decreasing inflammatory cell infiltration, alleviating focal consolidation, and reducing hydroxyproline content [41]. HSYA might inhibit the proliferation and migration of MRC-5 cells induced by TGF-β1 through targeting TGFβR2, modulating ECM deposition, and slowing the progression of pulmonary fibrosis [39]. Notably, HSYA exhibited protective effects comparable to dexamethasone in mitigating pulmonary fibrosis and inflammatory infiltration [97].

### 4.6 Cancer

HSYA has emerged as a multi-target candidate in oncology, with preclinical evidence supporting its efficacy against hepatocellular carcinoma (HCC), colorectal cancer (CRC), glioma, and non-small cell lung cancer (NSCLC) through modulation of proliferative, inflammatory, and metastatic pathways. Recent reviews [8] comprehensively summarize its broad anticancer mechanisms, including apoptosis induction, autophagy regulation, and tumor microenvironment modulation. This section deliberately focuses on representative studies to avoid redundancy.

In HCC, HSYA inhibited angiogenesis in tumor-bearing mice via suppressing the ERK/MAPK and NF-κB signaling pathway. While less effective than the anti-tumor drug sorafenib in reducing tumor size, HSYA exhibited a remarkable protection on immune organs in a dose-dependent manner [21]. In addition, it promoted apoptosis in HepG2 cells by inhibiting the p38MAPK signaling pathway [98]. In CRC, HSYA inhibited the proliferation and migration of HCT116 cells through activating the PPARγ/PTEN/Akt pathway. However, PPARγ antagonists only partially diminished the effects, suggesting that additional mechanisms require further investigation [30]. In gliomas, the inhibitory effect of HSYA was associated with the DNA damage response mediated, broadening its potential applications to neurological tumors [99]. In NSCLC, HSYA inhibited the proliferation, migration, and inflammatory responses of A549 and H1299 cells while promoting apoptosis. Its therapeutic efficacy in inflammation-mediated NSCLC was achieved through suppressing the PI3K/Akt/mTOR and ERK/MAPK signaling pathways [100]. HSYA also induced ferroptosis in osteosarcoma cells and mitigated the Warburg effect by inhibiting the HIF-1α/hexokinase 2 (HK2) and SLC7A11 pathways, suggesting its therapeutic potential in tumor suppression [101]. Current anti-tumor research on HSYA requires further in-depth exploration. Overreliance on single-pathway analyses neglects crosstalk with tumor microenvironment components. Future studies should focus on elucidating underlying mechanisms and expanding into other relevant areas.

### 4.7 Other diseases

Beyond the aforementioned diseases, HSYA also exhibits regulatory effects on other pathological conditions. Although research in these areas remains limited, it holds potential for further expanding the therapeutic applications of HSYA. In sepsis, HSYA mitigated CD4^+^ T lymphocyte apoptosis and suppressed systemic cytokine storms, collectively ameliorating the progression of sepsis [102]. In a sepsis model induced by cecal ligation and puncture, HSYA alleviated liver, lung, and kidney injury by regulating multi-organ metabolic pathways, thereby improving animal survival rates [103]. In osteoarthritis, HSYA inhibited NF-κB and MAPK signaling pathways to suppress inflammatory cytokines expression at the mRNA level, protecting chondrocytes from inflammatory damage [22]. In endometritis, it attenuated *Staphylococcus aureus* (*S. aureus*)-induced uterine damage by disrupting TLR2-mediated NF-κB/MAPK cascades, reducing inflammatory infiltration and preserving uterine tissue integrity [104]. In the renal I/R model, HSYA preserved kidney function and reduced apoptosis by inhibiting CCR4-associated signal pathways [105].

## 5 The clinical application of HSYA

The transition of HSYA from preclinical studies to clinical applications represents a pivotal milestone in validating its therapeutic potential and addressing long-term translational challenges. To date, clinical trials have demonstrated encouraging results, particularly in acute IS. In a phase II clinical trial (Registration No.: ChiCTR2000029608), patients received intravenous administration of HSYA for 14 days. Results confirmed that the proportion of patients achieving a modified Rankin scale score ≤ 1 at 90 days post-treatment was significantly higher than that of the control group. Medium-dose (50 mg/day) and high-dose (75 mg/day) HSYA treatment also showed greater proportions of favorable outcomes based on the National Institute of Health Stroke Scale (NIHSS) and Barthel index (BI) scores. Furthermore, these groups demonstrated greater improvement in blood stasis syndrome scores and exhibited good treatment tolerance across all tested doses during the follow-up period. Based on these findings, 50 and 75 mg/day are recommended for the phase III trial [4]. This phase II trial (Registration No.: ChiCTR2000038701) has been officially registered to further evaluate the efficacy and safety of HSYA in treating acute IS. However, the results have not yet been released, and no related publications are currently available. Additional clinical investigations have explored allergic reactions to HSYA (Registration No.: CTR20132888) and assessed its pharmacokinetics and safety following single or multiple intravenous infusions (Registration No.: ChiCTR2300072306). However, these findings remain to be further tracked and reported. Collectively, these clinical trials have accelerated the clinical application of HSYA.

Furthermore, HSYA is a key active constituent in Danhong injection, Xuebijing injection, and Safflower yellow injection. These traditional Chinese medicine formulations have not only been approved by the National Medical Products Administration, China, but also been incorporated into expert consensus guidelines for cardiovascular diseases, IS, and sepsis. Their successful clinical application confirms that HSYA-containing preparations can meet the stringent requirements for injectable therapeutic agents in terms of safety, quality control, and clinical efficacy. This successful clinical precedent offers a valuable reference for developing HSYA as a single-component drug, particularly with regard to efficacy validation, safety evaluation, and regulatory pathways. It further underscores the feasibility of developing standardized and well-characterized HSYA formulations under Good Manufacturing Practice (GMP) conditions. It also demonstrates that plant-derived injections could be effectively integrated into modern medical practice when supported by high-quality evidence.

## 6 Current limitations in HSYA research and development

Although early studies have demonstrated that HSYA exerts significant cardioprotective and neuroprotective effects, its broad pharmacological potential remains hindered in clinical translation due to several challenges. Key obstacles include intrinsic physicochemical limitations, inconsistent dosing strategies, and incomplete mechanistic elucidation. In particular, unresolved issues related to stability, pharmacokinetics, and crosstalk among signaling pathways continue to impede clinical advancement. A systematic summary of these deficiencies and challenges could not only help mitigate therapeutic risks but also lay the groundwork for improving clinical efficacy and guiding future applications.

### 6.1 Intrinsic physicochemical and biological properties limitations

**Fig. 4:**
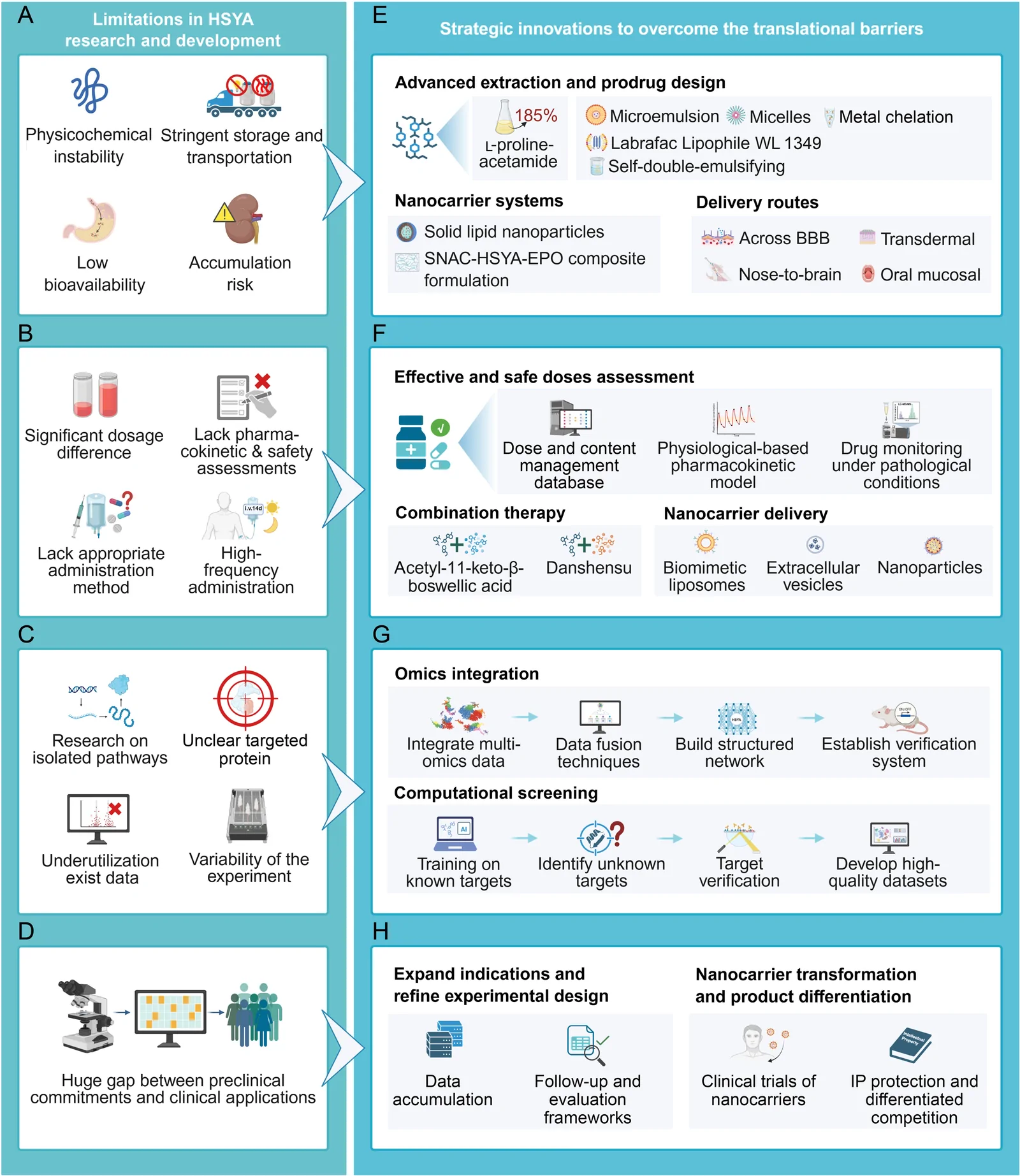
The current limitations and strategic innovations of hydroxysafflor yellow A (HSYA). (A) Intrinsic physicochemical and biological properties limitations. (B) Bottleneck of medication in the existing research. (C) Lack of in-depth mechanism exploration. (D) Shortage of impetus for clinical translation. (E) To enhance stability and bioavailability. (F) To standardize the dosage and administration protocol. (G) To elucidate mechanistic complexity. (H) To promote extensive clinical application. SNAC: *n*-(8-(2-hydroxybenzoyl) amino) sodium octanoate; EPO: Eudragit® EPO; BBB: blood-brain barrier; IP: intellectual property.

The therapeutic potential of HSYA is constrained by its inherent physicochemical instability and low bioavailability. Despite its high water solubility, its poor lipid solubility impairs membrane permeability, particularly for intracellular targets. Following oral administration, HSYA traverses the lipid bilayer of the intestinal epithelial cell membrane inefficiently, leading to low oral bioavailability. HSYA has a short half-life and low bioavailability, making it challenging to maintain therapeutic blood concentrations. Therefore, repeated dosing or increased dosage is often required. Oral dosing exhibits a shorter half-life compared to intravenous administration, further reinforcing reliance on the latter. Other drug administration routes also fail to effectively cross biological barriers such as the BBB, thus limiting their therapeutic utility. Although its water solubility facilitates gastric dissolution, HSYA is prone to pH-induced degradation under both acidic and basic conditions, reducing the availability of active ingredients. Additionally, it is also sensitive to light and heat, which necessitates stringent manufacturing and storage and transport conditions and causes additional cost. Moreover, such sensitivity complicates drug administration and contributes to fluctuations in drug concentration. Given that the kidneys serve as the primary elimination organ of HSYA, the risk of accumulation remains unclear in patients with renal insufficiency following repeated administrations or dose escalation. Therefore, addressing these physicochemical and biological constraints is essential for unlocking its full potential. Targeted strategies such as prodrug design, nanocarrier-based delivery, and advanced administration route engineering are urgently needed to overcome these limitations and improve clinical viability (Fig. 4A).

### 6.2 Bottleneck of medication in the existing research

The clinical translation of HSYA has been hindered by substantial inconsistencies in dosing regimens and administration routes. First, the drug dosage administered in different disease models varies considerably, with a lack of standardized dosing protocols. *In vitro*, the concentrations range from 1 to 100 μM. *In vivo*, doses vary from 2 mg/kg in a 6-hydroxydopamine-induced PD model to 250 mg/kg in an HFD-induced obesity model. Even within the same disease category (e.g., hepatic disorders), therapeutic doses differ markedly. Preclinical studies have confirmed the dose-dependent effect, yet pharmacokinetic and safety data remain incomplete. For example, repeated administering at 60 or 180 mg/kg for 90 days prolonged clotting times. The higher dose increases liver indices without inducing histopathological changes, but induces renal tubular injury [106,107]. Beyond the aforementioned adverse reactions, there remains a knowledge gap regarding the existence of other potential long-term effects of HSYA. Important safety concerns have not been thoroughly investigated, such as whether accumulation occurs in specific organs, particularly the kidneys. A comprehensive assessment of safety pharmacological data regarding immune regulation and genotoxicity is currently lacking. Striking a balance between the effective dose and the safe dose remains a critical focus for future research. Pathological conditions such as blood stasis after HSYA pharmacokinetics complicate dosage control. Gender-based pharmacokinetic differences have also been observed, suggesting the need for demographic-specific dosing strategies [11].

Second, current administration routes for animal models are primarily limited to oral, intravenous, and intraperitoneal delivery. Oral administration offers good compliance for chronic diseases such as atherosclerosis and obesity. However, it is hindered by first-pass metabolism and gastrointestinal degradation, necessitating higher doses and leading to variable absorption. Intravenous injection enables rapid and precise drug delivery but carries side effects related to peak concentration. This invasive method poses operational difficulties for chronic disease management. Intraperitoneal injection offers moderate pharmacokinetics and balances efficacy, but dose conversion challenges are still encountered. None of these administration methods has achieved effective lesion-site targeting, and the concentration of HSYA remaining in the lesion remains relatively low. Furthermore, given that HSYA demonstrates multi-target effects across various systems (cardiovascular, nervous, and digestive systems, etc.), it raises the possibility of off-target effects. Currently, there is insufficient data regarding its biological distribution and organ-specific safety profile. Selecting a more appropriate administration method is thus an influential factor affecting the pharmacological effects of HSYA.

Third, multiple dosing remains a necessary intervention strategy due to the rapid clearance of HSYA, which increases operational complexity and reduces patient compliance. Therefore, exploring ways to reduce dosing frequency while maintaining therapeutic effects is essential for streamlining treatment protocols. Future research should focus on defining standardized dosing protocols while minimizing dosage and frequency without compromising efficacy, and exploring delivery systems that enhance tissue targeting (Fig. 4B).

### 6.3 Lack of in-depth mechanism exploration

Although the multifaceted effects of HSYA (e.g., NF-κB and HIF-1α pathways) have been extensively demonstrated, the exploration of its underlying mechanisms remains fragmented. First, current research predominantly focuses on isolated signaling pathways while neglecting the broader regulatory role of HSYA in the disease microenvironment and systemic organ interactions. There is a lack of studies investigating pathway crosstalk, compensatory feedback, and upstream regulatory networks, which are critical for understanding drug resistance and variability in efficacy. For instance, in tumor intervention studies, the inhibitory effects of HSYA on tumor cell proliferation and migration have been highlighted. However, the crosstalk between HSYA and the tumor microenvironment, systemic immunity, or epigenetic regulatory factors has been largely overlooked. These interactions are critical for a comprehensive understanding of HSYA. It is imperative to further elucidate the therapeutic mechanisms of HSYA more systematically and holistically.

Second, some studies have confirmed direct interactions between HSYA and specific proteins such as HIF-1α and SIRT1 using molecular docking techniques. Nevertheless, their precise roles in therapeutic outcomes remain unclear. Identifying these targets and clarifying their functional roles is an urgent priority for advancing mechanistic understanding.

Third, it is also unknown whether the consistent pharmacological effects (anti-inflammatory and antioxidant, etc.) across diverse models share common upstream mechanisms or operate independently. In particular, omics-based approaches including transcriptomics and metabolomics remain underutilized, limiting the comprehensive integration of mechanistic insights within the context of disease.

Finally, many mechanistic studies originate from small-scale, single-laboratory experiments, often lacking adequate sample sizes, blinding, or randomization. These methodological shortcomings may undermine the reproducibility and credibility. A more rigorous and systematic evaluation is needed.

In summary, the current state of mechanism research on HSYA reveals notable gaps. A more comprehensive and systematic approach to mechanism elucidation across various disease models is essential for promoting successful clinical translation (Fig. 4C).

### 6.4 Shortage of impetus for clinical translation

Clinical research on HSYA remains limited, with most trials lacking long-term follow-up data and adequately powered sample sizes. At present, investigations have focused mainly on cardiovascular and CNS diseases, whereas potential benefits in cancer, fibrosis, and metabolic disorders are largely unexplored. This gap likely reflects both the need for stronger preclinical evidence and challenges in trial design for these conditions. Moreover, current trials often overlook inherent pharmacokinetic challenges, such as poor oral bioavailability. Although intravenous administration is effective, frequent dosing due to rapid renal clearance reduces compliance and cost-effectiveness. Furthermore, the variability of disease-specific pharmacokinetics highlights the need for pathologically adjusted dosing.

Clinical trial designs also tend to prioritize symptom-based outcomes over biological endpoints. For example, while preclinical models indicate that the efficacy of HSYA in addressing obesity arises from the modulation of the gut microbiota, no clinical trials to date have incorporated microbiota analysis to validate this hypothesis. In addition, while HSYA is a key component of widely used multi-component formulations such as Danhong injection and Xuebijing injection, it has not yet been directly compared with these agents in clinical settings. Its current evaluation remains largely confined to a limited number of preclinical studies [61,108], which restricts a more comprehensive understanding of its individual therapeutic potential. While promising preclinical evidence supports the use of nanoparticles loaded with HSYA, this delivery approach has yet to be investigated in clinical trials. In summary, accelerating HSYA's journey from bench to bedside will require integrating robust preclinical evidence with well-designed clinical trials that address dosing, delivery, mechanistic validation, and broader therapeutic indications (Fig. 4D).

## 7 Strategic innovations to overcome the translational barriers of HSYA

To bridge the gap between the preclinical potential and clinical application of HSYA, an integrated multidisciplinary approach is essential for its development. This section outlines the strategic approaches to overcome the current limitations of HSYA and to harmonize dosing regimens. These advancements aim to unlock its full therapeutic potential while ensuring its efficacy, safety, and adaptability across diverse clinical settings.

### 7.1 To enhance stability and bioavailability

The therapeutic potential of HSYA is constrained by its inherent physicochemical instability, poor lipid solubility, and rapid systemic clearance. These limitations collectively hinder its clinical application. Addressing these challenges requires a multifaceted strategy integrating advanced extraction and prodrug design, nanocarrier system engineering, and delivery route optimization (Fig. 4E).

#### 7.1.1 Advanced extraction and prodrug design

More advanced extraction techniques have been developed to improve yield and enhance bioavailability. L-proline-acetamide, a natural deep eutectic solvent, is recognized as an effective solvent for the extraction of HSYA (yielding 32.83 mg/g). The bioavailability of HSYA obtained via this method is approximately 183.5% of that from traditional aqueous extract. The implementation of this extraction method substantially improves both the extraction efficiency and utilization [109]. Prodrug design could further enhance the stability and bioavailability of HSYA by chemically masking labile functional groups. For instance, a microemulsion of HSYA markedly enhanced its oral bioavailability, showing an approximately 1937% increase compared with a control solution in bile duct-nonligated rats [110]. Likewise, self-double-emulsifying drug delivery systems have improved the apparent permeability and increased intestinal uptake by 2.17-fold [111]. Labrafac Lipophile WL 1349 improved oral bioavailability through bile-mediated emulsification [112]. HSYA exhibits superior stability against pH and temperature in Xuebijing injection compared to its aqueous solution, likely due to the protective effect of micelles. This phenomenon could serve as a viable strategy for enhancing the stability of HSYA [9]. Metal chelation leverages the phenolic hydroxyl groups of HSYA to form stable complexes with metal ions, offering a feasible strategy for delivering hydrophilic natural products. For instance, HSYA-iron complexes enhance stability, achieve excellent biocompatibility, and have a potent antioxidant capacity [113].

#### 7.1.2 Nanocarrier systems

Various nanocarrier-based formulations have also been developed to improve pharmacokinetic properties and enhance therapeutic efficacy. Nanoparticle encapsulation provides a dual benefit by enhancing both the bioavailability and stability of HSYA, protecting it from photodegradation and enabling controlled release. Solid lipid nanoparticles (SLNs) encapsulating HSYA maintain stability for up to 10 days at 4 °C or 30 °C and increase the oral bioavailability by 3.97-fold, demonstrating superior efficacy in reducing cerebral infarction volume compared to aqueous HSYA solutions [114]. A composite material (*n*-(8-(2-hydroxybenzoyl) amino) sodium octanoate (SNAC)-HSYA-Eudragit® EPO (EPO)) was developed through the joint preparation of oral penetration enhancer SNAC, EPO, and HSYA. This composite formulation integrates the advantages of SNAC and EPO to enhance absorption by improving permeability and lipophilicity, enabling sustained *in vivo* release [115].

#### 7.1.3 Delivery routes

In addition to modifying drug properties and carriers, choosing an optimal delivery route is crucial, particularly for targeting the brain and other organs with restrictive barriers. Nanomaterials could be employed to improve the ability of HSYA to cross the BBB. For example, HSYA-hydroxypropyl-β-cyclodextrin phospholipid complex water-in-oil nanoemulsions (HC@HMC) enhanced oral absorption and blood concentration. It achieved BBB penetration with a peak concentration of 23.46 ng/mL, which is 1.15 times higher than that of HSYA alone [116]. Co-administration of HSYA with the BBB modulator Lexiscan further increased the accumulation of HSYA in the ischemic brain tissue and achieved a more pronounced neuroprotective effect [117]. Emerging administration routes have demonstrated promising therapeutic outcomes, such as intranasal or mucosal delivery. For example, the nose-to-brain delivery approach could bypass the BBB, enabling the effective transport of natural products to the target brain [118]. Likewise, transdermal and oral mucosal administration can sustain drug absorption while avoiding hepatic first-pass metabolism. These delivery strategies may be particularly advantageous for the long-term management of CNS diseases, chronic inflammatory, or metabolic disorders. Although these approaches have not yet been investigated in HSYA, their success with other natural compounds warrants further exploration.

Collectively, the integration of extraction and prodrug design, nanocarrier systems, and optimized delivery routes provides a comprehensive framework to overcome the physicochemical and pharmacokinetic challenges. These innovations pave the way for the development of clinically viable HSYA formulations with enhanced stability, bioavailability, and therapeutic efficacy.

### 7.2 To standardize the dosage and administration protocol

Inconsistent drug administration patterns and management-related challenges have impeded the translational potential of HSYA. In order to establish an evidence-based drug administration regimen, a multi-level approach needs to be adopted (Fig. 4F).

#### 7.2.1 Effective and safe doses assessment

A comprehensive evaluation of the effective and safe doses of HSYA is required. This includes the formulation of standardized safety assessment criteria and the establishment of a dose and content management database [119]. A physiologically based pharmacokinetic model can help reconcile *in vivo* and *in vitro* differences, aiding early risk assessment and human dose prediction. It addresses deficiencies in existing studies and enables the assessment of the safe medication range [120,121]. Given that the kidney serves as the metabolic organ for HSYA, applying a physiology-based kidney model allows integration of renal anatomical and physiological characteristics with the unique properties of HSYA, such as its hydrophilicity and low fat solubility. This facilitates capturing dynamic systems and drug interactions, thus advancing pharmacokinetic investigations [122]. Moreover, therapeutic drug monitoring using liquid chromatography tandem mass spectrometry (LC-MS/MS) detection supports personalized drug administration under pathological conditions. As previously noted, the pharmacokinetics of HSYA vary under different pathological conditions. Therefore, avoiding toxicity while maintaining efficacy is crucial. Currently, there are few high-quality studies on the pharmacokinetics of HSYA under pathological conditions. Including pharmacokinetic evaluations under these conditions as part of therapeutic effect assessments could promote the better application of HSYA.

#### 7.2.2 Combination therapy

Combining HSYA with other drugs can both potentiate therapeutic efficacy and reduce HSYA dosage requirements. For instance, the combination of HSYA and acetyl-11-keto-β-boswellic acid, an active ingredient extracted from *Boswellia serrata* (*B. serrata*), reduced the HSYA dosage from 100 to 50 mg/kg while enhancing antioxidant activity to counteract myocardial injury [36]. Similarly, co-administration of HSYA and Danshensu decreased the HSYA dosage from 70 to 35 mg/kg, synergistically producing excellent antioxidant and anti-apoptotic effects for cardiac protection [123]. While most current research focuses primarily on evaluating whether combination therapies outperform HSYA monotherapy, it is undeniable that drug combinations reduce HSYA dosages, potentially enhancing safety by alleviating toxic side effects. Nonetheless, further research is required to assess possible drug-drug interactions and emergent safety concerns.

#### 7.2.3 Nanocarrier delivery

Nanocarriers offer an effective strategy for improving the management of HSYA doses by enhancing tissue targeting, prolonging circulation time, and decreasing administration frequency. HSYA-loaded biomimetic liposomes exhibit favorable stability and sustained-release properties. They deliver HSYA to atherosclerotic plaques, prolonging systemic retention and facilitating localized drug accumulation. In an atherosclerosis model, administering HSYA-loaded biomimetic liposomes twice weekly produced superior therapeutic outcomes compared with conventional four-week or daily administration regimens [124]. Key strategies for maximizing delivery efficiency include extending systemic circulation time, reducing non-specific uptake via surface modification, and protecting HSYA from degradation while enabling controlled release through carrier encapsulation. Furthermore, the application of extracellular vesicles [125] and nanoparticles [126] in the field of natural products offers valuable references for the nanocarrier delivery of HSYA. These nanocarriers have targeted delivery and high loading capacity, while facilitating precise dose control of HSYA.

These strategies collectively contribute to standardizing HSYA dosages in disease applications, achieving excellent therapeutic efficacy at lower doses and simplifying administration frequencies, ultimately promoting clinical translation.

### 7.3 To elucidate mechanistic complexity

The therapeutic effects of HSYA are mediated through multi-target interactions. However, mechanistic studies remain fragmented and lack systematic integration. An integrated pharmacological framework is therefore essential to unravel its polypharmacology. The adoption of a multi-center approach plays a crucial role in facilitating an in-depth investigation of the underlying mechanism [127]. To effectively address this complexity, it is essential to develop a collaborative strategy integrating omics analysis, artificial intelligence (AI)-assisted computational prediction, and experimental validation, establishing a closed-loop framework from discovery to mechanistic elucidation (Fig. 4G).

#### 7.3.1 Omics integration

Omics technologies provide a powerful mean for systematically dissecting the mechanism of HSYA. Spatiotemporal multi-omics provide a holistic view of the systemic effects of HSYA, such as transcriptomics, proteomics, and metabolomics [128]. By leveraging the distinct yet complementary features of multi-omics approaches, their integration enables a comprehensive understanding of HSYA's effects across multiple biological levels. These layers range from transcriptional regulation, protein expression, and metabolic alterations to the dynamic changes in upstream and downstream mechanisms [129]. Furthermore, multi-omics integration can differentiate between genuine potential targets and incidental variations. Subsequently, gene prioritization and pathway enrichment analysis can be performed on multi-omics datasets through data fusion techniques such as directional *P*-value merging (DPM) [130], generating a structured network linking HSYA to specific molecular targets, biological pathways, and disease phenotypes. This approach can further reveal compensatory mechanisms and inter-pathway crosstalk, thereby deepening mechanistic insight into HSYA's role in complex disease. In parallel, this approach facilitates the investigation of whether additional products with pharmacological effects are generated during HSYA metabolism and bioransformation [131].

Once network construction is complete, a more comprehensive set of validation experiments is needed. For instance, it is crucial to analyze downstream pathways by integrating animal-level gene knockout/knock-in studies with cell-level knockdown/overexpression experiments. Such approaches will facilitate a deeper understanding of the mechanism through which HSYA binds to its target protein and influences disease progression [132]. However, multi-omics data primarily reflect secondary or downstream biological effects. Identifying direct targets of HSYA necessitates orthogonal approaches. Techniques such as limited proteolytic MS (LIP-MS) can map HSYA-protein interactions at the proteome level, yielding direct binding evidence [133]. Integrating the indirect inferences derived from multi-omics analyses with the direct target identifications enables the construction of a highly reliable drug-target interaction map. This dual-layer strategy not only strengthens mechanistic understanding but also elucidates the protein targets of HSYA, broadening the scope of its therapeutic applications. Multi-omics integration captures the comprehensive molecular response of the biological system to HSYA. This approach enables precise identification of interconnected pathways and minimizes overreliance on isolated signaling cascades.

It is important to emphasize that the robustness of multi-omics driven HSYA-targeted disease network is highly dependent on the quality and reliability of the underlying experimental data. Future verification research should prioritize methodological rigor, including adequate sample size, appropriate randomization, blinding procedures, and well-defined biological endpoints. These considerations are essential for ensuring data accuracy, reducing bias, and improving reproducibility.

#### 7.3.2 Computational screening

After constructing a network through multi-omics collaboration, AI tools can further expand and refine the network by predicting novel or less evident targets. Graph neural networks (GNNs) are capable of learning from the established omics networks by incorporating features such as differential gene expression, protein-protein interactions, and disease associations. By training on the relationships of known drug targets, GNNs can predict additional targets of HSYA. For example, GraphBAN, a GNN-based framework, has been developed to provide an effective solution for predicting interactions between entirely unseen compounds and proteins [134]. Another advancement involves the integration of disease-specific profiling with target screening capabilities through deep neural network models. For example, training models using specific AD biomarkers allows for the identification of natural products capable of modulating AD-related pathological processes [135]. These examples highlight complementary strategies for AI-assisted target discovery. When applied to HSYA-targeted disease networks, it holds promise for systematically identifying uncharacterized targets. Beyond network-based AI models, pharmacophore modeling and structure-based deep learning approaches can be employed to assess the interaction potential of HSYA with proteins of structural or functional similarity [136]. These AI-driven platforms do not replace omics research, but rather integrate and enhance multi-omics networks into dynamic, predictive analytical systems.

High-confidence targets predicted through these approaches must undergo rigorous experimental validation. Methods such as surface plasmon resonance (SPR) and cellular thermal shift assay (CETSA) can directly measure HSYA-target binding affinity and confirm target engagement in a cellular context [132]. This process completes the inference-validation cycle and improves the predictive performance of the model through feedback learning. By integrating multi-omics networks with AI-driven dynamics, researchers can prioritize high-value targets of HSYA, offering a cost-effective strategy to decode the polypharmacology of HSYA. Moreover, leveraging AI technology to consolidate resources [137] and generate high-quality datasets from existing research will facilitate the development of a comprehensive, data-driven understanding of the research landscape and prospects of HSYA.

### 7.4 To promote extensive clinical application

Although HSYA exhibits significant pharmacological potential, its clinical translation remains limited. Bridging the gap between preclinical research and clinical application requires a comprehensive translational strategy (Fig. 4H).

#### 7.4.1 Expand indications and refine experimental design

The issue of limited diseases in HSYA clinical trials needs to be addressed. Future clinical protocols should consider combination therapies involving HSYA, particularly when supported by strong preclinical evidence. Subsequently, clinical indications can be expanded based on accumulating data. Current symptom-oriented approaches often overlook the integration of relevant biomarkers. Therefore, multi-omics analyses should be incorporated to better capture the multi-target effects of HSYA. Clinical studies should enhance methodological rigor by adopting adequate sample sizes, multicenter recruitment, randomization, blinding, and long-term follow-up procedures to monitor outcomes. Given the variability in dosing across preclinical models and individualized metabolic differences, clinical research should prioritize the development of personalized treatment regimens for patients with renal or hepatic impairments. Furthermore, preclinical studies should be recognized through more comprehensive and standardized evaluation frameworks.

#### 7.4.2 Nanocarrier translation and product differentiation

Nanocarrier-based delivery systems have shown promising preclinical results, but require translation into early-phase clinical trials with thorough pharmacokinetic and safety assessments. Lessons can be drawn from clinical trial experiences of ursolic acid nanocarrier systems (Registration No.: CTR20230593), which offer a valuable reference for overcoming pharmacokinetic limitations and enhancing the clinical utility. From a translational perspective, it is essential to also consider the intellectual property (IP) strategy and business development plan of HSYA. As a natural product with a publicly disclosed chemical structure, HSYA is no longer eligible for compound structure patent protection. It should focus its patent strategy on areas such as formulation optimization, drug delivery systems, and combination therapies. HSYA is already a key constituent in several approved multi-component formulations, which presents a challenge in achieving product differentiation. Differentiated development can be achieved through innovative administration routes, such as nasal-to-brain and transdermal delivery, expanded indications, and reinforced IP protection. Implementing these strategies will unlock the full therapeutic potential of HSYA across diverse pathologies, supporting robust and patient-centered clinical outcomes.

## 8 Future prospects

HSYA exemplifies the typical challenges encountered in the development of natural product drugs, including their immense potential and inherent complexity. Its broad-spectrum therapeutic effects, including anti-inflammatory, cardiovascular protective, and neuroprotective properties, underscore its capacity to engage in multi-target mechanisms. However, this multi-pharmacological profile also complicates mechanistic clarity, as evidenced by fragmented studies focusing solely on isolated pathways without integrating crosstalk or systemic factors. Similarly, clinical trials have not fully leveraged preclinical findings, reflecting a disconnect between mechanism discovery and therapeutic application.

What further complicates these challenges is the intrinsic vulnerability of HSYA, characterized by its physicochemical instability and pharmacokinetic limitations, which present significant obstacles to its clinical application. While strategic innovations such as nanoparticle encapsulation and prodrug design have shown promise in enhancing stability and bioavailability, long-term safety and scalability remain unresolved challenges. Moreover, the absence of standardized dosing regimens remains a significant limitation, exemplified by the wide dose range observed in various disease models. This underscores the need for physiology-based pharmacokinetic modeling and therapeutic drug monitoring to optimize regimens tailored to specific pathologies and patient subgroups.

The way forward necessitates a systematic pharmacological framework that integrates multiple disciplines and transcends traditional methodologies. Realizing the full therapeutic potential of HSYA hinges on the integration of three critical aspects: i) overcoming performance limitations via drug design or advanced drug delivery systems; ii) standardizing application through precise dosing regimens and well-defined procedures; iii) and bridging translational barriers by in-depth mechanistic investigations with clinical development pathways. Meanwhile, the recent elucidation of the biosynthetic pathway for HSYA offers additional opportunities to scale production and accelerate industrialization.

**Fig. 5:**
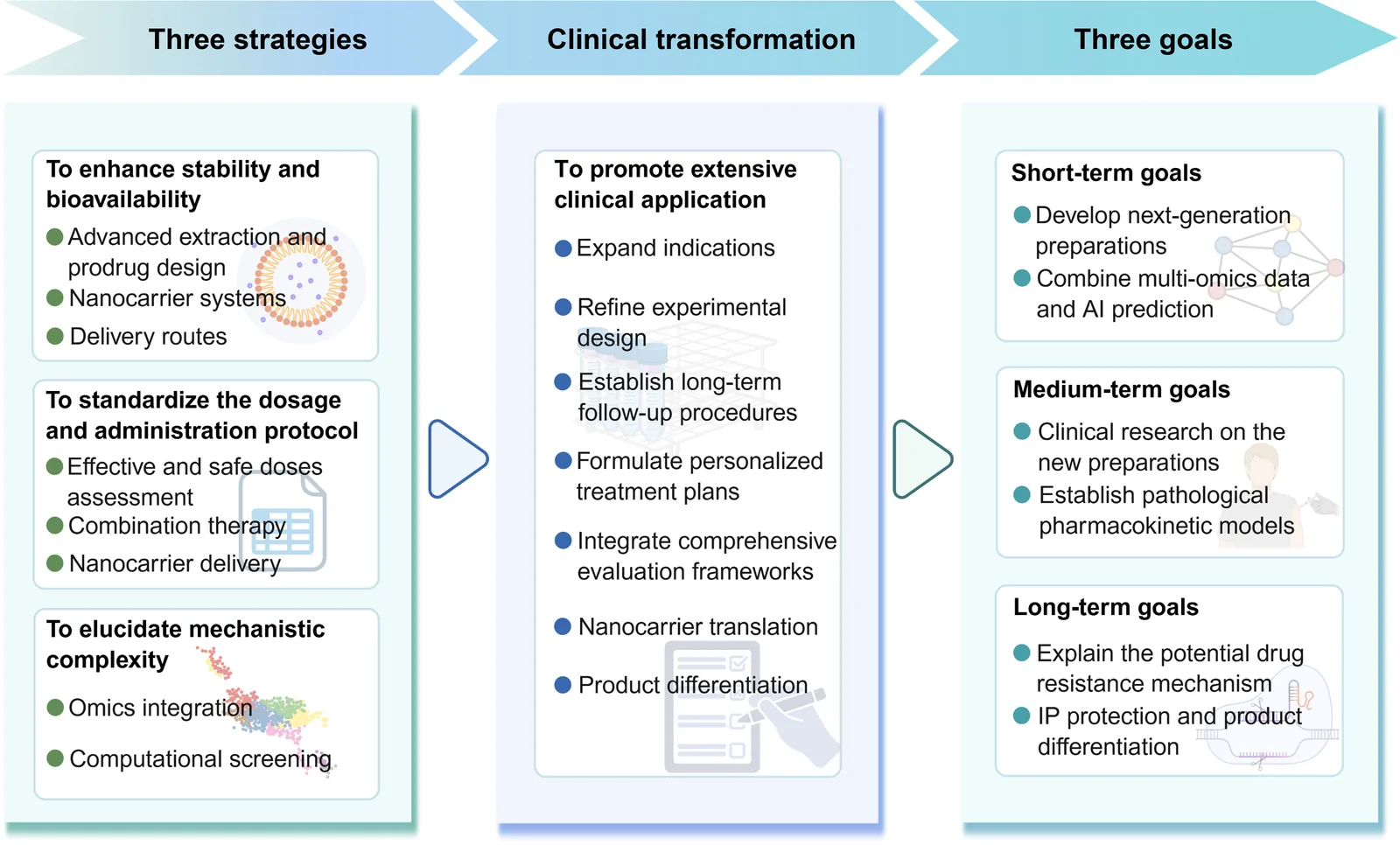
From strategy to goal in the development of hydroxysafflor yellow A (HSYA). AI: artificial intelligence; IP: intellectual property.

To accelerate the translation of HSYA into a clinically viable therapeutic agent, it is essential to develop a structured and feasibility-driven roadmap that outlines clear strategic priorities (Fig. 5). In the short term, efforts should focus on bridging the gap between preclinical and clinical research. Concurrently, drawing upon nanoscale formulation strategies (e.g., liposomes and SLNs) successfully applied to other natural products, the development of next-generation preparations should be prioritized to enhance the bioavailability and stability of HSYA. Furthermore, integrating existing omics datasets with AI-assisted target discovery can help refine its pharmacological profile and identify novel therapeutic targets. Medium-term priorities involve conducting the first clinical study using optimized next-generation preparation delivery systems, focusing on oral or sustained-release delivery systems to improve patient compliance. In parallel, efforts should focus on establishing standardized pharmacokinetic models across various pathological conditions, such as hepatic or renal impairment. In the long term, advanced technologies such as clustered regularly interspaced short palindromic repeats (CRISPR)-based functional genomics and single-cell transcriptomics can be employed to explain more complex pharmacology. Following the development of the next-generation preparations and the completion of clinical trials, the application of these technologies facilitates the discovery of complex regulatory networks and drug resistance [138]. Meanwhile, due attention should be given to IP protection and product differentiation. Ultimately, establishing a multi-phase pipeline that integrates formulation engineering, mechanistic studies, and early-phase clinical trials is critical to transitioning HSYA from empirical application to evidence-based, precision medicine.

## 9 Conclusions

In conclusion, the translation of HSYA exemplifies the common challenges faced by numerous natural products in clinical translation. The outlined strategies hold broad reference value. The advancement of HSYA necessitates the interdisciplinary collaboration that integrates the empirical knowledge of traditional medicine with the methodical analytical frameworks of modern pharmacology. Through a systematic strategy grounded in nanostructure engineering, systemic pharmacology, computer technology guidance, and clinical translation, HSYA is poised to serve as a model for the translation and development of other natural products.

## CRediT authorship contribution statement

**Haonan Zhu:** Writing – original draft. **Xiao Ni:** Formal analysis. **Zhe Yu:** Conceptualization. **Si Liang:** Formal analysis. **Yi Qiu:** Visualization. **Jun Huang:** Supervision. **Tao Tang:** Supervision. **Yang Wang:** Project administration. **En Hu:** Writing – review & editing, Project administration. **Teng Li:** Writing – review & editing, Project administration.

## Declaration of competing interest

The authors declare that they have no known competing financial interests or personal relationships that could have appeared to influence the work reported in this paper.

## References

[1] Xue X., Deng Y., Wang J. (2021). Hydroxysafflor yellow A, a natural compound from *Carthamus tinctorius* L with good effect of alleviating atherosclerosis. Phytomedicine.

[2] Cheng C., Ren C., Li M. (2024). Pharmacologically significant constituents collectively responsible for anti-sepsis action of XueBiJing, a Chinese herb-based intravenous formulation. Acta Pharmacol. Sin..

[3] Feng X., Li Y., Wang Y. (2019). Danhong injection in cardiovascular and cerebrovascular diseases: pharmacological actions, molecular mechanisms, and therapeutic potential. Pharmacol. Res..

[4] Hu M., Zhou Z., Zhou Z. (2020). Effect and safety of hydroxysafflor yellow A for injection in patients with acute ischemic stroke of blood stasis syndrome: a phase II, multicenter, randomized, double-blind, multiple-dose, active-controlled clinical trial, Chin. J. Integr. Med..

[5] Zhao F., Wang P., Jiao Y. (2020). Hydroxysafflor yellow A: a systematical review on botanical resources, physicochemical properties, drug delivery system, pharmacokinetics, and pharmacological effects. Front. Pharmacol..

[6] Feng Z., He J., Jiang J. (2013). NMR solution structure study of the representative component hydroxysafflor yellow A and other quinochalcone C-glycosides from *Carthamus tinctorius*. J. Nat. Prod..

[7] Wang Z., Wang H., Chang G. (2025). Elucidation of the biosynthetic pathway of hydroxysafflor yellow A. Nat. Commun..

[8] Wang Y., An J., Zhou J. (2024). Hydroxysafflor yellow A: a natural pigment with potential anticancer therapeutic effect. Front. Pharmacol..

[9] Pu W., Zhang H., Wang M. (2017). Superior stability of hydroxysafflor yellow A in xuebijing injection and the associated mechanism. Molecules.

[10] Zhao X., Gu S., Wang X. (2025). The safety, tolerability, and pharmacokinetics of active ingredients from hydroxysafflor yellow A in healthy Chinese volunteers. Clin. Pharmacol. Drug Dev..

[11] Li C., Yin J., Zhang J. (2015). Pharmacokinetic profiles of hydroxysafflor yellow A following intravenous administration of its pure preparations in healthy Chinese volunteers. J. Ethnopharmacol..

[12] Wen A., Yang J., Jia Y. (2008). A rapid and sensitive liquid chromatography–tandem mass spectrometry (LC–MS/MS) method for the determination of hydroxysafflor yellow A in human plasma: application to a pharmacokinetic study. J. Chromatogr. B.

[13] Zhang X., Shen D., Feng Y. (2021). Pharmacological actions, molecular mechanisms, pharmacokinetic progressions, and clinical applications of hydroxysafflor yellow A in antidiabetic research. J. Immunol. Res..

[14] Chu D., Liu W., Huang Z. (2006). Pharmacokinetics and excretion of hydroxysafflor yellow A, a potent neuroprotective agent from safflower, in rats and dogs. Planta Med..

[15] Tian Y., Yang Z., Li Y. (2010). Pharmacokinetic comparisons of hydroxysafflower yellow A in normal and blood stasis syndrome rats. J. Ethnopharmacol..

[16] Jin Y., Wu L., Tang Y. (2016). UFLC-Q-TOF/MS based screening and identification of the metabolites in plasma, bile, urine and feces of normal and blood stasis rats after oral administration of hydroxysafflor yellow A. J. Chromatogr. B Anal. Technolog. Biomed. Life Sci..

[17] Balaji S., Ahmed M., Lorence E. (2018). NF-κB signaling and its relevance to the treatment of mantle cell lymphoma. J. Hematol. Oncol..

[18] Yu H., Lin L., Zhang Z. (2020). Targeting NF-κB pathway for the therapy of diseases: mechanism and clinical study. Signal Transduct. Targeted Ther..

[19] Sun S. (2011). Non-canonical NF-κB signaling pathway. Cell Res..

[20] Guo X., Zheng M., Pan R. (2018). Hydroxysafflor yellow A suppresses platelet activating factor-induced activation of human small airway epithelial cells. Front. Pharmacol..

[21] Yang F., Li J., Zhu J. (2015). Hydroxysafflor yellow A inhibits angiogenesis of hepatocellular carcinoma via blocking ERK/MAPK and NF-κB signaling pathway in H22 tumor-bearing mice. Eur. J. Pharmacol..

[22] Hu Z., Xie Z., Tang Q. (2018). Hydroxysafflor yellow A (HSYA) targets the NF-κB and MAPK pathways and ameliorates the development of osteoarthritis. Food Funct..

[23] Wang W., Liu M., Fu X. (2024). Hydroxysafflor yellow A ameliorates alcohol-induced liver injury through PI3K/Akt and STAT3/NF-κB signaling pathways. Phytomedicine.

[24] Masoud G.N., Li W. (2015). HIF-1α pathway: role, regulation and intervention for cancer therapy. Acta Pharm. Sin. B.

[25] Ge C., Meng D., Peng Y. (2024). The activation of the HIF-1α-VEGFA-Notch1 signaling pathway by Hydroxysafflor yellow A promotes angiogenesis and reduces myocardial ischemia-reperfusion injury. Int. Immunopharmacol..

[26] Ge C., Peng Y., Li J. (2023). Hydroxysafflor yellow A alleviates acute myocardial ischemia/reperfusion injury in mice by inhibiting ferroptosis via the activation of the HIF-1α/SLC7A11/GPX4 signaling pathway. Nutrients.

[27] Wei R., Song L., Miao Z. (2022). Hydroxysafflor yellow A exerts neuroprotective effects via HIF-1α/BNIP3 pathway to activate neuronal autophagy after OGD/R. Cells.

[28] Yu L., Wei J., Liu P. (2022). Attacking the PI3K/Akt/mTOR signaling pathway for targeted therapeutic treatment in human cancer. Semin. Cancer Biol..

[29] Feng X., Du M., Li S. (2023). Hydroxysafflor yellow A regulates lymphangiogenesis and inflammation via the inhibition of PI3K on regulating AKT/mTOR and NF-κB pathway in macrophages to reduce atherosclerosis in ApoE^-/-^ mice. Phytomedicine.

[30] Su D., Lv C. (2021). Hydroxysafflor yellow A inhibits the proliferation, migration, and invasion of colorectal cancer cells through the PPARγ/PTEN/Akt signaling pathway. Bioengineered.

[31] Li T., Zhang L., Cheng M. (2024). Metabolomics integrated with network pharmacology of blood-entry constituents reveals the bioactive component of Xuefu Zhuyu decoction and its angiogenic effects in treating traumatic brain injury. Chin. Med..

[32] Rojo de la Vega M., Chapman E., Zhang D.D. (2018). NRF2 and the hallmarks of cancer. Cancer Cell.

[33] Fão L., Mota S.I., Rego A.C. (2019). Shaping the Nrf2-ARE-related pathways in Alzheimer's and Parkinson's diseases. Ageing Res. Rev..

[34] Wang Y., Han K., Li Z. (2022). Protective effect of hydroxysafflor yellow A on renal ischemia --reperfusion injury by targeting the Akt-Nrf2 axis in mice. Exp. Ther. Med..

[35] Ni B., Zhou D., Jing Y. (2018). Hydroxysafflor yellow A protects against angiotensin II-induced hypertrophy. Mol. Med. Rep..

[36] Chen M., Wang M., Yang Q. (2022). Antioxidant effects of hydroxysafflor yellow A and acetyl-11-keto-β-boswellic acid in combination on isoproterenol-induced myocardial injury in rats. Int. J. Mol. Med..

[37] Kim D.H., Sung M., Park M.S. (2024). Galectin 3-binding protein (LGALS3BP) depletion attenuates hepatic fibrosis by reducing transforming growth factor-β1 (TGF-β1) availability and inhibits hepatocarcinogenesis. Cancer Commun. Lond. Engl..

[38] Kim K.K., Sheppard D., Chapman H.A. (2018). TGF-β1 signaling and tissue fibrosis. Cold Spring Harbor Perspect. Biol..

[39] Pan R., Zhang Y., Zheng M. (2017). Hydroxysafflor yellow A suppresses MRC-5 cell activation induced by TGF-β1 by blocking TGF-β1 binding to TβRII. Front. Pharmacol..

[40] Xue X., Li Y., Zhang S. (2024). Hydroxysafflor yellow A exerts anti-fibrotic and anti-angiogenic effects through miR-29a-3p/PDGFRB axis in liver fibrosis. Phytomedicine.

[41] Jin M., Wu Y., Wang L. (2016). Hydroxysafflor yellow A attenuates bleomycin-induced pulmonary fibrosis in mice. Phytother Res..

[42] Xu C., Wang L., Fozouni P. (2020). SIRT1 is downregulated by autophagy in senescence and ageing. Nat. Cell Biol..

[43] Fangma Y., Zhou H., Shao C. (2021). Hydroxysafflor yellow A and anhydrosafflor yellow B protect against cerebral ischemia/reperfusion injury by attenuating oxidative stress and apoptosis via the silent information regulator 1 signaling pathway. Front. Pharmacol..

[44] Qin X., Chen J., Zhang G. (2020). Hydroxysafflor yellow A exerts anti-inflammatory effects mediated by SIRT1 in lipopolysaccharide-induced microglia activation. Front. Pharmacol..

[45] Yao M., Liu Y., Meng D. (2025). Hydroxysafflor yellow A attenuates the inflammatory response in cerebral ischemia-reperfusion injured mice by regulating microglia polarization per SIRT1-mediated HMGB1/NF-κB signaling pathway. Int. Immunopharmacol..

[46] Rong J., Li C., Zhang Q. (2023). Hydroxysafflor yellow A inhibits endothelial cell ferroptosis in diabetic atherosclerosis mice by regulating miR-429/SLC7A11. Pharm. Biol..

[47] Liu Y., Piao J., He J. (2024). Hydroxysafflor yellow A, a natural food pigment, ameliorates atherosclerosis in ApoE^-/-^ mice by inhibiting the SphK1/S1P/S1PR3 pathway. Food Sci. Nutr..

[48] Wang H., Liu J., Yang Y. (2016). Hydroxy-safflower yellow A inhibits the TNFR1-mediated classical NF-κB pathway by inducing shedding of TNFR1. Phytother Res..

[49] Ye J., Wang M., Wang R. (2020). Hydroxysafflor yellow A inhibits hypoxia/reoxygenation-induced cardiomyocyte injury via regulating the AMPK/NLRP3 inflammasome pathway. Int. Immunopharmacol..

[50] Zou J., Wang N., Liu M. (2018). Nucleolin mediated pro-angiogenic role of Hydroxysafflor yellow A in ischaemic cardiac dysfunction: Post-transcriptional regulation of VEGF-A and MMP-9. J. Cell Mol. Med..

[51] Wei G., Yin Y., Duan J. (2017). Hydroxysafflor yellow A promotes neovascularization and cardiac function recovery through HO-1/VEGF-A/SDF-1α cascade. Biomed. Pharmacother..

[52] Zhao F., Cheng W., Wu D. (2025). Hydroxysafflor yellow A ameliorates myocardial ischemia/reperfusion injury by promoting MDH1-mediated mitochondrial metabolic homeostasis. Phytomedicine.

[53] Ye J., Lu S., Wang M. (2020). Hydroxysafflor yellow A protects against myocardial ischemia/reperfusion injury via suppressing NLRP3 inflammasome and activating autophagy. Front. Pharmacol..

[54] Zhou D., Ding T., Ni B. (2019). Hydroxysafflor yellow A mitigated myocardial ischemia/reperfusion injury by inhibiting the activation of the JAK2/STAT1 pathway. Int. J. Mol. Med..

[55] Ji X., Lei C., Kong S. (2024). Hydroxy-safflower yellow A mitigates vascular remodeling in rat pulmonary arterial hypertension. Drug Des. Dev. Ther..

[56] Feng J., Guo J., Yan J. (2023). Luhong formula and Hydroxysafflor yellow A protect cardiomyocytes by inhibiting autophagy. Phytomedicine.

[57] Huang W., Yao W., Weng Y. (2023). Hydroxysafflor yellow A inhibits the hyperactivation of rat platelets by regulating the miR-9a-5p/SRC axis. Arch. Biochem. Biophys..

[58] Li Y., Gu J., Ge J. (2024). HSYA ameliorates venous thromboembolism by depleting the formation of TLR4/NF-κB pathway-dependent neutrophil extracellular traps. Int. Immunopharmacol..

[59] Wang L., Cui X., He J. (2021). Hydroxysafflor yellows alleviate thrombosis and acetaminophen-induced toxicity *in vivo* by enhancing blood circulation and poison excretion. Phytomedicine.

[60] Chen J., Ren C., Yao C. (2023). Identification of the natural *Chalcone* glycoside hydroxysafflor yellow A as a suppressor of P53 overactivation-associated hematopoietic defects. MedComm.

[61] Guo Y., Yang J., Cao S. (2021). Effect of main ingredients of Danhong Injection against oxidative stress induced autophagy injury via miR-19a/SIRT1 pathway in endothelial cells. Phytomedicine.

[62] Yang J., Wang R., Cheng X. (2020). The vascular dilatation induced by Hydroxysafflor yellow A (HSYA) on rat mesenteric artery through TRPV4-dependent calcium influx in endothelial cells. J. Ethnopharmacol..

[63] Wang Z., Liu Z., Hao Y. (2025). Hydroxysafflor yellow A promotes the proliferation and differentiation of endogenous neural stem cells with neural regenerative effects in ischemic stroke. Phytomedicine.

[64] Huang P., Wu S., Wang N. (2021). Hydroxysafflor yellow A alleviates cerebral ischemia reperfusion injury by suppressing apoptosis via mitochondrial permeability transition pore. Phytomedicine.

[65] Cui Q., Ma Y., Yu H. (2021). Systematic analysis of the mechanism of hydroxysafflor yellow A for treating ischemic stroke based on network pharmacology technology. Eur. J. Pharmacol..

[66] Wu Y., Yin L., Wang Z. (2025). Hydroxysafflor yellow A inhibits neuronal ferroptosis and ferritinophagy in ischemic stroke. Biochim. Biophys. Acta BBA Mol. Basis Dis..

[67] Chen S., Sun M., Zhao X. (2019). Neuroprotection of hydroxysafflor yellow A in experimental cerebral ischemia/reperfusion injury via metabolic inhibition of phenylalanine and mitochondrial biogenesis. Mol. Med. Rep..

[68] Li Y., Liu X., Zhang P. (2022). Hydroxysafflor yellow a blocks HIF-1α induction of NOX2 and protects ZO-1 protein in cerebral microvascular endothelium. Antioxidants.

[69] Yu L., Duan Y., Zhao Z. (2018). Hydroxysafflor yellow a (HSYA) improves learning and memory in cerebral ischemia reperfusion-injured rats via recovering synaptic plasticity in the hippocampus. Front. Cell. Neurosci..

[70] Chen G., Li C., Zhang L. (2022). Hydroxysafflor yellow A and anhydrosafflor yellow B alleviate ferroptosis and parthanatos in PC12 cells injured by OGD/R. Free Radic. Biol. Med..

[71] Pei J., Fan L., Nan K. (2017). HSYA alleviates secondary neuronal death through attenuating oxidative stress, inflammatory response, and neural apoptosis in SD rat spinal cord compression injury. J. Neuroinflammation.

[72] Lai Z., Li C., Ma H. (2023). Hydroxysafflor yellow A confers neuroprotection against acute traumatic brain injury by modulating neuronal autophagy to inhibit NLRP3 inflammasomes. J. Ethnopharmacol..

[73] Wang Y., Zhang C., Peng W. (2016). Hydroxysafflor yellow A exerts antioxidant effects in a rat model of traumatic brain injury. Mol. Med. Rep..

[74] Hu E., Li T., Li Z. (2023). Metabolomics reveals the effects of hydroxysafflor yellow A on neurogenesis and axon regeneration after experimental traumatic brain injury. Pharm. Biol..

[75] Hou J., Wang C., Zhang M. (2020). Safflower yellow improves the synaptic structural plasticity by ameliorating the disorder of glutamate circulation in Aβ1-42-induced AD model rats. Neurochem. Res..

[76] Ren M., Zhang M., Zhang X. (2022). Hydroxysafflor yellow A inhibits Aβ1–42-induced neuroinflammation by modulating the phenotypic transformation of microglia via TREM2/TLR4/NF-κB pathway in BV-2 cells. Neurochem. Res..

[77] Zhang N., Xing M., Wang Y. (2014). Hydroxysafflor yellow A improves learning and memory in a rat model of vascular dementia by increasing VEGF and NR1 in the hippocampus, Neurosci. Bull. (Arch. Am. Art).

[78] Yang X., Li Y., Chen L. (2020). Protective effect of hydroxysafflor yellow A on dopaminergic neurons against 6-hydroxydopamine, activating anti-apoptotic and anti-neuroinflammatory pathways. Pharm. Biol..

[79] Yu H., Bai Q., Guo J. (2024). Elucidating hydroxysafflor yellow a's multi-target mechanisms against alcoholic liver disease through integrative pharmacology. Phytomedicine.

[80] Hou X., Zhang Z., Ma Y. (2022). Mechanism of hydroxysafflor yellow A on acute liver injury based on transcriptomics. Front. Pharmacol..

[81] Sun L., Yang R., Hou X. (2025). Regulation of endoplasmic reticulum stress and liver injury by hydroxysafflor yellow a through mir-222-3p-pten-wnt axis. Eur. J. Pharmacol..

[82] Wu L., Dong X., Sun W. (2025). Hydroxysafflor yellow A alleviates oxidative stress and inflammatory damage in the livers of mice with nonalcoholic fatty liver disease and modulates gut microbiota. Front. Pharmacol..

[83] Li Y., Shi Y., Sun Y. (2017). Restorative effects of hydroxysafflor yellow A on hepatic function in an experimental regression model of hepatic fibrosis induced by carbon tetrachloride. Mol. Med. Rep..

[84] Wang C.Y., Liu Q., Huang Q.X. (2013). Activation of PPARγ is required for hydroxysafflor yellow A of *Carthamus tinctorius* to attenuate hepatic fibrosis induced by oxidative stress. Phytomedicine.

[85] Chen J., Pan M., Wang J. (2023). Hydroxysafflor yellow A protects against colitis in mice by suppressing pyroptosis via inhibiting HK1/NLRP3/GSDMD and modulating gut microbiota. Toxicol. Appl. Pharmacol..

[86] Feng Z., Zhou P., Wu X. (2022). Hydroxysafflor yellow A protects against ulcerative colitis via suppressing TLR4/NF-κB signaling pathway. Chem. Biol. Drug Des..

[87] Liu J., Yue S., Yang Z. (2018). Oral hydroxysafflor yellow A reduces obesity in mice by modulating the gut microbiota and serum metabolism. Pharmacol. Res..

[88] Lyu X., Yan K., Hu W. (2024). Safflower yellow and its main component hydroxysafflor yellow A alleviate hyperleptinemia in diet-induced obesity mice through a dual inhibition of the GIP-GIPR signaling axis. Phytother Res..

[89] Lyu X., Yan K., Xu H. (2022). Intragastric safflower yellow and its main component HSYA improve leptin sensitivity before body weight change in diet-induced obese mice. Naunyn-Schmiedebergs Arch Pharmacol.

[90] Yan K., Wang X., Pan H. (2020). Safflower yellow and its main component HSYA alleviate diet-induced obesity in mice: possible involvement of the increased antioxidant enzymes in liver and adipose tissue. Front. Pharmacol..

[91] Yan K., Wang X., Zhu H. (2020). Safflower yellow improves insulin sensitivity in high-fat diet-induced obese mice by promoting peroxisome proliferator-activated receptor-γ2 expression in subcutaneous adipose tissue. J. Diabetes Investig..

[92] Li Y., Zheng D., Shen D. (2020). Protective effects of two safflower derived compounds, kaempferol and hydroxysafflor yellow A, on hyperglycaemic stress-induced podocyte apoptosis via modulating of macrophage M1/M2 polarization. J. Immunol. Res..

[93] Min F., Sun H., Wang B. (2020). Hepatoprotective effects of hydroxysafflor yellow A in D-galactose-treated aging mice. Eur. J. Pharmacol..

[94] Kong J., Sun S., Min F. (2022). Integrating network pharmacology and transcriptomic strategies to explore the pharmacological mechanism of Hydroxysafflor yellow A in delaying liver aging. Int. J. Mol. Sci..

[95] Wang Y., Guo Y., Wen P. (2020). Three ingredients of safflower alleviate acute lung injury and inhibit NET release induced by lipopolysaccharide. Mediat. Inflamm..

[96] Jin M., Xue C., Wang Y. (2019). Protective effect of hydroxysafflor yellow A on inflammatory injury in chronic obstructive pulmonary disease rats, Chin. J. Integr. Med..

[97] Jin M., Wang L., Wu Y. (2018). Protective effect of hydroxysafflor yellow A on bleomycin-induced pulmonary inflammation and fibrosis in rats. Chin. J. Integr. Med..

[98] Zhang J., Li J., Song H. (2019). Hydroxysafflor yellow A suppresses angiogenesis of hepatocellular carcinoma through inhibition of p38 MAPK phosphorylation. Biomed. Pharmacother..

[99] Tang D., Huang T., Tian Q. (2021). MYC/NBS1-mediated DNA damage response is involved in the inhibitory effect of hydroxysafflor yellow A on glioma cells. Drug Des. Dev. Ther..

[100] Jiang M., Zhou L., Xu N. (2019). Hydroxysafflor yellow A inhibited lipopolysaccharide-induced non-small cell lung cancer cell proliferation, migration, and invasion by suppressing the PI3K/AKT/mTOR and ERK/MAPK signaling pathways, Thorac. Cancer.

[101] Zhu Y., Yang L., Yu Y. (2024). Hydroxysafflor yellow A induced ferroptosis of Osteosarcoma cancer cells by HIF-1α/HK2 and SLC7A11 pathway. Oncol. Res..

[102] Wang J., Wang P., Gui S. (2017). Hydroxysafflor yellow a attenuates the apoptosis of peripheral blood CD4^+^ T lymphocytes in a murine model of sepsis. Front. Pharmacol..

[103] Pan B., Chen F., Shen S. (2025). Hydroxysafflor yellow A modulation of metabolite networks and inhibition of JAK2/STAT1 pathway in sepsis. Sci. Rep..

[104] He S., Wang X., Liu Z. (2021). Hydroxysafflor yellow A inhibits *Staphylococcus aureus*-induced mouse endometrial inflammation via TLR2-mediated NF-kB and MAPK pathway. Inflammation.

[105] Wang R., Jia D., Zhang W. (2025). Hydroxysafflower yellow A mitigates renal ischemia-reperfusion injury by inhibiting the CCR4-mediated apoptosis pathway. Phytomedicine.

[106] Liu Z., Li C., Li M. (2004). The subchronic toxicity of hydroxysafflor yellow A of 90 days repeatedly intraperitoneal injections in rats. Toxicology.

[107] Yu L., Jin Z., Li M. (2022). Protective potential of hydroxysafflor yellow A in cerebral ischemia and reperfusion injury: an overview of evidence from experimental studies. Front. Pharmacol..

[108] Ren C., Yao R., Wang L. (2021). Comparison of effects of Xuebijing injection and its component hydroxysafflor yellow A on coagulation and survival rates of septic rats. Zhonghua Wei Zhong Bing Ji Jiu Yi Xue.

[109] Tong X., Yang J., Zhao Y. (2021). Greener extraction process and enhanced *in vivo* bioavailability of bioactive components from *Carthamus tinctorius* L. by natural deep eutectic solvents. Food Chem..

[110] Qi J., Zhuang J., Wu W. (2011). Enhanced effect and mechanism of water-in-oil microemulsion as an oral delivery system of hydroxysafflor yellow A. Int. J. Nanomed..

[111] Lv L., Tong C., Lv Q. (2012). Enhanced absorption of hydroxysafflor yellow A using a self-double-emulsifying drug delivery system: *in vitro* and *in vivo* studies. Int. J. Nanomed..

[112] Wang S., Sun M., Ping Q. (2008). Enhancing effect of Labrafac Lipophile WL 1349 on oral bioavailability of hydroxysafflor yellow A in rats. Int. J. Pharm..

[113] Tang Q., Yi Y., Chen Y. (2022). A green and highly efficient method to deliver hydrophilic polyphenols of *Sal*via *miltiorrhiza* and *Carthamus tinctorius* for enhanced anti-atherosclerotic effect via metal-phenolic network. Colloids Surf. B Biointerfaces.

[114] Zhao B., Gu S., Du Y. (2018). Solid lipid nanoparticles as carriers for oral delivery of hydroxysafflor yellow A. Int. J. Pharm..

[115] Bao D., Xie X., Cheng M. (2022). Hydroxy-safflower yellow A composites: an effective strategy to enhance anti-myocardial ischemia by improving intestinal permeability. Int. J. Pharm..

[116] Zhang Y., Zhong C., Wang Q. (2023). Nanoemulsions of hydroxysafflor yellow A for enhancing physicochemical and *in vivo* performance. Int. J. Mol. Sci..

[117] Tan L., Wang Y., Jiang Y. (2020). Hydroxysafflor yellow A together with blood–brain barrier regulator lexiscan for cerebral ischemia reperfusion injury treatment. ACS Omega.

[118] Maisto N., Mango D. (2024). Nose to brain strategy coupled to nano vesicular system for natural products delivery: focus on synaptic plasticity in Alzheimer's disease. J. Pharm. Anal..

[119] Enioutina E.Y., Job K.M., Krepkova L.V. (2020). How can we improve the safe use of herbal medicine and other natural products? A clinical pharmacologist mission. Expert Rev. Clin. Pharmacol..

[120] Santos L.G.A., Jaiswal S., Chen K. (2025). Real-world application of physiologically based pharmacokinetic models in drug discovery. Drug Metab. Dispos..

[121] Isoherranen N. (2025). Physiologically based pharmacokinetic modeling of small molecules: how much progress have we made?. Drug Metab. Dispos..

[122] Huang W., Bowman C., Yin M. (2025). A review of physiologically based pharmacokinetic modeling of renal drug disposition. Drug Metab. Dispos..

[123] Hu T., Wei G., Xi M. (2016). Synergistic cardioprotective effects of Danshensu and hydroxysafflor yellow A against myocardial ischemia-reperfusion injury are mediated through the Akt/Nrf2/HO-1 pathway. Int. J. Mol. Med..

[124] Li Y., Yang A., Sun Y. (2023). Hydroxysafflor yellow A-loaded biomimetic liposomes alleviate HHcy-induced atherosclerosis by regulating methylation related autophagy. Mater. Des..

[125] Cheng H., Su G., Wu Y. (2024). Extracellular vesicles in anti-tumor drug resistance: mechanisms and therapeutic prospects. J. Pharm. Anal..

[126] Zheng H., Chen Y., Luo W. (2025). Integration of active ingredients from traditional Chinese medicine with nano-delivery systems for tumor immunotherapy. J. Nanobiotechnol..

[127] Tu Y., Dai G., Chen Y. (2025). Emerging target discovery strategies drive the decoding of therapeutic power of natural products and further drug development: a case study of celastrol. Explorations.

[128] Chen C., Wang J., Pan D. (2023). Applications of multi-omics analysis in human diseases. MedComm.

[129] O'Connor L.M., O'Connor B.A., Bin Lim S. (2023). Integrative multi-omics and systems bioinformatics in translational neuroscience: a data mining perspective. J. Pharm. Anal..

[130] Slobodyanyuk M., Bahcheli A.T., Klein Z.P. (2024). Directional integration and pathway enrichment analysis for multi-omics data. Nat. Commun..

[131] Luo F., Yu Z., Zhou Q. (2022). Multi-omics-based discovery of plant signaling molecules. Metabolites.

[132] Zhu Y., Ouyang Z., Du H. (2022). New opportunities and challenges of natural products research: when target identification meets single-cell multiomics. Acta Pharm. Sin. B.

[133] Van Leene C., Van Moortel L., De Bosscher K. (2025). Exploring protein conformations with limited proteolysis coupled to mass spectrometry. Trends Biochem. Sci..

[134] Hadipour H., Li Y., Sun Y. (2025). GraphBAN: an inductive graph-based approach for enhanced prediction of compound-protein interactions. Nat. Commun..

[135] Wu T., Lin R., Cui P. (2024). Deep learning-based drug screening for the discovery of potential therapeutic agents for Alzheimer's disease. J. Pharm. Anal..

[136] Shrestha R., Fajardo J.E., Fiser A. (2021). Residue-based pharmacophore approaches to study protein-protein interactions. Curr. Opin. Struct. Biol..

[137] Zhang Y., Wang Y. (2023). Recent trends of machine learning applied to multi-source data of medicinal plants. J. Pharm. Anal..

[138] Wang S., Gao C., Zheng Y. (2022). Current applications and future perspective of CRISPR/Cas9 gene editing in cancer. Mol. Cancer.

